# Hydrolysis of ionic liquid–treated substrate with an *Iocasia fonsfrigidae* strain SP3-1 endoglucanase

**DOI:** 10.1007/s00253-023-12918-1

**Published:** 2024-01-08

**Authors:** Sobroney Heng, Sawannee Sutheeworapong, Chinnapong Wangnai, Verawat Champreda, Akihiko Kosugi, Khanok Ratanakhanokchai, Chakrit Tachaapaikoon, Ruben Michael Ceballos

**Affiliations:** 1 School of Bioresources and Technology, King Mongkut’s University of Technology Thonburi, Bangkok, 10150 Thailand; 2 Department of Molecular and Cell Biology, University of California, Merced, CA 95343 USA; 3 Systems Biology and Bioinformatics Laboratory, Pilot Plant Development and Training Institute, King Mongkut’s University of Technology Thonburi, Bangkok, 10150 Thailand; 4 Pilot Plant Development and Training Institute, King Mongkut’s University of Technology Thonburi, Bangkok, Thailand; 5 National Center for Genetic Engineering and Biotechnology, 113 Thailand Science Park, Paholyothin Road Klong Luang, Pathumthani, 12120 Thailand; 6 Biological Resources and Post-Harvest Division, Japan International Research Center for Agricultural Sciences, Ibaraki, Japan; 7 Excellent Center of Enzyme Technology and Microbial Utilization, Pilot Plant Development and Training Institute, King Mongkut’s University of Technology Thonburi, Bangkok, 10150 Thailand; 8 Quantitative Systems Biology Program, University of California, Merced, CA 95343 USA

**Keywords:** Endoglucanase, Glycoside hydrolase family 12, Salt tolerance, Ionic liquids, *Iocasia fonsfrigidae* strain SP3-1, Enzyme platform

## Abstract

**Abstract:**

Recently, we reported the discovery of a novel endoglucanase of the glycoside hydrolase family 12 (GH12), designated IfCelS12A, from the haloalkaliphilic anaerobic bacterium *Iocasia fonsfrigidae* strain SP3-1, which was isolated from a hypersaline pond in the Samut Sakhon province of Thailand (ca. 2017). IfCelS12A exhibits high substrate specificity on carboxymethyl cellulose and amorphous cellulose but low substrate specificity on b-1,3;1,4-glucan. Unlike some endoglucanases of the GH12 family, IfCelS12A does not exhibit hydrolytic activity on crystalline cellulose (i.e., Avicel™). High-Pressure Liquid Chromatography (HPLC) and Thin Layer Chromatography (TLC) analyses of products resulting from IfCelS12-mediated hydrolysis indicate mode of action for this enzyme. Notably, IfCelS12A preferentially hydrolyzes cellotetraoses, cellopentaoses, and cellohexaoses with negligible activity on cellobiose or cellotriose. Kinetic analysis with cellopentaose and barely b-d-glucan as cellulosic substrates were conducted. On cellopentaose, IfCelS12A demonstrates a 16-fold increase in activity (*K*_*M*_ = 0.27 mM; *k*_*cat*_ = 0.36 s^−1^; *k*_*cat*_*/K*_*M*_ = 1.34 mM^−1^ s^−1^) compared to the enzymatic hydrolysis of barley b-d-glucan (*K*_*M*_: 0.04 mM, *k*_*cat*_: 0.51 s^−1^, *k*_*cat*_*/K*_*M*_ = 0.08 mM^−1^ s^−1^). Moreover, IfCelS12A enzymatic efficacy is stable in hypersaline sodium chlorids (NaCl) solutions (up to 10% NaCl). Specifically, IfCel12A retains notable activity after 24 h at 2M NaCl (10% saline solution). IfCelS12A used as a cocktail component with other cellulolytic enzymes and in conjunction with mobile sequestration platform technology offers additional options for deconstruction of ionic liquid–pretreated cellulosic feedstock.

**Key points:**

• *IfCelS12A from an anaerobic alkaliphile Iocasia fronsfrigidae shows salt tolerance*

• *IfCelS12A in cocktails with other enzymes efficiently degrades cellulosic biomass*

• *IfCelS12A used with mobile enzyme sequestration platforms enhances hydrolysis*

**Supplementary Information:**

The online version contains supplementary material available at 10.1007/s00253-023-12918-1.

## Introduction

Optimizing processes and developing innovative approaches for the efficient deconstruction of cellulosic biomass toward producing high-value products (e.g., biofuel) is a robust area of research (Lynd et al. 1991; Ceballos et al. 2014, 2015; Ceballos 2017; Fatema et al. 2022). Lignocellulose is a principal component of forestry waste, bamboo, switch grass, and crop residues, including sugarcane bagasse; sorghum stover; corn pericarp or stover; and wheat straw and rice straw (Kostas et al. 2017). Rice straw is an abundant, renewable cellulosic biomass resource in Thailand (Jusakulvijit et al. 2021). Indeed, 18–29 megatons (MT) of rice straw is currently burned annually as crop waste in Thailand (Jusakulvijit et al. 2021). It is estimated that 731 (MT) of rice straw is destroyed annually as an unwanted crop residue worldwide (Hu et al. 2021). In addition to losing potential energy resources, large-scale burning of crop residues in fields often produces 2.5 mm particulate matter (i.e., PM2.5), which is detrimental to human health (Radabutra et al. 2021).

As a lignocellulose material, rice straw is composed of lignin, hemicellulose, and cellulose at percent compositions of 5–24%, 19–27%, and 32–47%, respectively (Kobkam et al. 2018). Like other lignocellulose feedstock, rice straw structure is complex with lignin providing a “glue-like” adhesion within and around intricate lattice of cellulose and hemicellulose (Ceballos 2017; Chen et al. 2021). Lignin is comprised of cross-linked polymers of phenolic monomers (Yang et al. 2022). Lignin in the cell wall of plants provides structural support, impermeability to solvents, and resistance to microbial attack (Bajpai 2016). Hemicellulose is a branched heteropolymer of hexose sugars, pentose sugars, and sugar acids (Saha 2003). Cellulose, a major component of lignocellulosic biomass (e.g., rice straw), is the main target for commercial applications. Cellulose is a straight-chain polymer of glucose monomers linked by β-1,4-glycosidic bonds (Kobkam et al. 2018). Cellulose can be degraded enzymatically to produce simpler, soluble (and fermentable) sugars (Giovannoni et al. 2020). Sugar released from the deconstruction of lignocellulose may be used to produce alcohol and other industrial chemicals (Baramee et al. 2017). However, accessing cellulose (and hemicellulose) from the lignocellulosic matrix is challenging (Ceballos 2017; Li et al. 2022). Indeed, this is a primary technical focus in developing feedstock degradation processes.

Many feedstock conversion processes employ stepwise treatment strategies to deconstruct lignocellulosic biomass, extract cellulose (and hemicellulose), and then decompose these polysaccharides to disaccharides and monosaccharides. Such processes will often include a chemical pretreatment with ammonia hydroxide, organic acids, sodium hydroxide, high temperature steam, or ionic liquids (IL) (Zhao et al. 2009; Kobkam et al. 2018; Phitsuwan et al. 2016; Kuntapa et al. 2021). To reduce the use of hazardous chemicals and to develop more environmentally safe processes, IL has garnered attention as a feedstock pretreatment option (Gunny and Arbain 2013). However, hypersaline solutions (e.g., 5–35% salt concentration) can be detrimental to subsequent enzyme-mediated steps in the feedstock degradation processes (Grewal et al. 2022). Specifically, hypersaline IL can inactivate enzymes by denaturing protein structure or interfering with the dynamics of enzyme-substrate binding (Salvador et al. 2010). Therefore, the benefits of using IL pretreatment of cellulosic biomass are overshadowed by the deleterious impacts on enzymes. Halophilic enzymes, however, are resistant to denaturation in IL.

In this study, we demonstrate that a recently reported endoglucanase, designated IfCelS12A (Heng et al. 2022), from the haloalkaliphile *Iocasia fonsfrigidae* strain SP3-1 (Heng et al. 2019, 2022) used in conjunction with a cocktail of enzymes from *Clostridium thermocellum* and in the presence of a mobile enzyme sequestration platform (MESP) (Mitsuzawa et al. 2009; Ceballos et al. 2014; Ceballos 2023) enhances enzymatic breakdown of multiple substrates including rice straw (RS) in IL. Our data further demonstrate that IfCelS12A has a mode of action distinct from other glycoside hydrolases within the GH12 family of enzymes. Cumulatively, this study suggests that IfCelS12A may provide unique contributions to the deconstruction of lignocellulosic biomass in enzyme cocktail-mediated degradation processes.

## Materials and methods

### Chemicals and media

Carboxymethyl cellulose (CMC) (400–800 cps), cellulose powder (Type 20), α-cellulose, Avicel PH101, cellobiose, glucose, glucose assay kit, and ionic liquids such as 1-butyl-3-methylimidazolium tetrafluoroborate (BMIM-BF4), 1-butyl-3-methylimidazolium acetate (BMIM-Ac), and 1-ethyl-3-methylimidazolium chloride (EMIM-Cl) were purchased from Sigma-Aldrich (St. Louis, MO, USA). Phosphoric acid-swollen cellulose (PASC) was prepared from Avicel PH101, as previously described by Zhang et al. (2006). All cello-oligosaccharide stocks (G_2_–G_6_) were purchased from Megazyme (Wicklow, Ireland). Bovine serum albumin (BSA) was purchased from Merck (Darmstadt, Germany). All other reagents were of analytical grade purity and obtained from similar commercial sources. 

### Bacterial strain and plasmids

The complete genome of *I. fonsfrigidae* strain SP3-1 was deposited in the National Center for Biotechnology Information (NCBI), under accession no. CP032760. Genomic DNA of *I. fonsfrigidae* strain SP3-1 was extracted from cell culture using a DNeasy Blood and tissue kit (Qiagen, Hilden, Germany) (Heng et al. 2019) and used as the source of chromosomal DNA. The plasmid pGEM®-T Easy (Promega, Madison, WI, USA) was used as a cloning vector, while the pET28a( +) vector (Novagen, Madison, WI, USA) was used for expression. Strains used for cloning and expression were *Escherichia coli* DH5a and BL21 (DE3) (Novagen, Madison, WI, USA), respectively. Strains were grown aerobically in Luria–Bertani broth (LB) at 37 **°**C with shaking at 200 rpm. Resulting *E. coli* transformants were grown in LB medium with antibiotics (100 μgmL^−1^of ampicillin or 50 μgmL^−1^ of kanamycin). Cell density was monitored by measuring optical density at 600 nm (OD_600nm_).

### Alignment and phylogenetic tree of IfCelS12A

Protein sequences for IfCelS12A and other glycoside hydrolase family 12 (GH12) enzymes extracted from NCBI were evaluated using the Clustal Omega multiple sequence alignment software tool https://www.ebi.ac.uk/Tools/msa/clustalo/ (accessed May 25, 2023). Phylogenetic trees for IfCelS12A (and other characterized GH12 members) were constructed using the neighbor-joining method with a bootstrap analysis of 1000 replicates in MEGA 11 (Tamura et al. 2021). IfCelS12A was compared to: *Gloeophyllum trabeum* (GtCel12A) strain ATCC 11539 (HQ163778), *Thermotoga naphthophila* (TnCel12B2) RKU-10 (ADA67369), *Aspergillus aculeatus* (AaXEG) (AAD02275), *Streptomyces lividans* (SlCelB2) (AAB71950), and *Bacillus licheniformis* (BlCel12A) (AAP44491).

### Analysis of genomic DNA and PCR amplification

The concentration of genomic DNA obtained from *I. fonsfrigidae* strain SP3-1 was determined by agarose gel and nanodrop (~ 100 ng μL^−1^). To amplify the *I. fonsfrigidae* strain SP3-1 gene encoding IfCelS12A, forward and reverse primers were used (10 pmol μL^−1^). These primers were designed with *Bam*HI and *Sal*I restriction sites, respectively (see underlined): 5′-GGATCCATGTTTATTGGCAGTTTT-3′ (forward) and 5′-GGGTCGACTTA TTGTTTATTTTTAATAA-3′ (reverse). PCR-based amplification conditions were as follows: 30 s at 95 °C followed by 35 cycles at 95 °C for 30 s, then 55 °C for 30 s, and 68 °C for 1 min with a final hold at 68 °C for 5min. The PCR product was purified using a PCR purification kit (Qiagen, Hilden, Germany). Successful amplification of the *IfCelS12A* gene was validated by checking sequences of resulting products via Illumina sequencing (Apical Scientific, Bangkok, Thailand).

### Cloning of endoglucanase gene IfCelS12A

Amplified DNA fragments were ligated into the pGEM®-T Easy vector and introduced into *E. coli* DH5α. Transformation was done by incubating cells with plasmids followed by selection on LB plates supplemented with 100 μg mL^−1^ ampicillin, 1 mM isopropyl β-d-1-thiogalactopyranoside (IPTG), and 80 μg mL^−1^ X-gal. Selected colonies were confirmed to harbor the plasmid by subsequent PCR analysis. The PCR fragment was digested with *Bam*HI and *Sal*I and ligated between the same restriction sites of the pET28a( +) vector, yielding pET28a( +)-*IfCelS12A*. The ligated construct was subsequently transformed into BL21 (DE3) *E. coli* expression cell line. Transformants were incubated and selected on LB plates containing 50 μg mL^−1^ kanamycin. Cloning was confirmed by PCR and DNA sequencing.

### Enzyme preparation

The *E. coli* BL21 (DE3) clone, harboring the plasmid pET28a( +)-*IfCelS12A*, was grown overnight at 37 °C in LB broth supplemented with 50 μg mL^−1^ kanamycin. The culture was then inoculated into a fresh LB medium (1:100 dilution) containing 50 μg mL^−1^ kanamycin and grown at 37 °C with 200 rpm shaking until optical density (OD_600 nm_) reached 0.6–0.8. Protein expression was subsequently induced with 0.5 mM IPTG. The culture was then incubated at 37 °C for 4 h. After incubation, cells were harvested by centrifugation (10 min, 6000 × g, 4 °C), washed twice with binding buffer (20 mM of imidazole), and resuspended in the same buffer. The cell suspension was broken by ultrasonication in an icebox for 30 min (on for 5 s, off for 5 s, 40 kHz, 250 W), and cell debris was removed by centrifugation at 10,000 × g for 20 min at 4°C. The following purification procedure was carried out at 4°C on a column packed with Ni Sepharose High-Performance affinity resin for His-tag proteins. After equilibrating the column with 10 column volumes of washed buffer (20 mM binding buffer), the supernatant of the cell extract was introduced to the HisTrap™ column (GE Healthcare, Little Chalfont, UK) for affinity purification. After sample loading, the column was washed with 10 column volumes of washing buffer, and then protein was eluted with 5 mL of 150 mM imidazole buffer. The eluate was concentrated by ultrafiltration (10 kDa cutoff). The expression and purification of heat shock protein cohesion-containing heat shock protein subunit β (HSPβ-coh) were carried out in a similar manner as previously described with modifications (Ceballos et al. 2014). BL21-CodonPlus (DE3)-RIL (Novagen, Madison, WI, USA) expressing HSPβ-coh were harvested by centrifugation at 7000 rpm for 10 min at 4 °C and stored at − 80 °C. Cell pellets were re-suspended in 50 mM Tris–HCl, containing 1 mM ethylenediaminetetraacetic acid (EDTA), lysozyme (0.1 mg mL^−1^), and protease inhibitor cocktail (0.5 mL g^−1^ of cell mass), pH 7. Cell lysis was performed by sonication, followed by centrifugation of suspension at 8,000 rpm for 30 min to remove cell debris. The cell-free lysate was treated with heat precipitation at 70 **°**C for 20 min and the nucleic acids were precipitated by adding 1.5% v/v of a 10% polyethyleneimine solution. After centrifugation at 8,000 rpm for 30 min, the supernatant was filtered using a 0.45 μm filter. The supernatant was applied to a HiPrep Q FF 16/10 column (Cytiva, Marlborough, MA, USA) equilibrated with 50 mM Tris–HCl, pH 7.5, containing 1 mM EDTA (buffer A) using an NGC Medium-Pressure Liquid Chromatography System (Bio-Rad, Hercules, CA, USA). The column was washed with five column volumes of buffer A, and bound protein was eluted with a 40-column volume gradient from 0 to 100% buffer A containing 1 M NaCl. Using a HiPrep 16/60 Sephacryl S-300 HR column (Cytiva, Marlborough, MA, USA), which was equilibrated with buffer A containing 300 mM NaCl, further purification was performed. Purified HSPβ-coh was buffer-exchanged to 20 mM Tris-maleate, pH 6, using an AMICON centrifugal filter (Sigma Millipore, St. Louis, MO, USA) before being used for MESP complex formation described below. Recombinant endoglucanase D (CelD), exoglucanase K (CelK), xylanase (XynA), and b-glucosidase (BglA) from *C. thermocellum* were purchased from C5-6 Technologies, LLC (Fitchburg, WI, USA). 

### Sodium dodecyl-sulfate polyacrylamide gel electrophoresis (SDS-PAGE) and zymogram analysis of IfCelS12A

The purity of the purified proteins was examined on a 10% PAG by the method of Laemmli (1970). After electrophoresis, the proteins were stained with Coomassie brilliant blue R-250. Molecular weight standards (i.e., protein ladder) was used as a high-molecular-weight calibration kit (Bio-Rad, Hercules, CA, USA). A CMCase zymogram was performed on sodium dodecyl sulfate (SDS) 10% polyacrylamide gel containing 0.1% CMC, as described previously (Phitsuwan et al. 2010). 

### MESP-enzyme complex assembly

The 18-mer nonameric double-ring MESP complex was assembled by incubating HSPb-coh subunits at 1 mg mL^−1^ in 20 mM Tris-maleate, pH 6.0 containing 1 mM ATP, and 50 mM MgCl_2_ at 4 °C overnight (Ceballos et al. 2014). To charge MESP with enzymes, 10 mM of MESP complex was incubated at room temperature for 15 min with 10 mM of IfCelS12A, CelD, CelK, XynA, and BglA enzymes in the presence of 20 mM Tris-maleate, pH 6, containing 1 mM ATP, 25 mM MgCl_2_, and 5 mM CaCl_2_. 

### Protein determination, enzyme assays, and product analysis of IfCelS12A

All experiments were done in triplicate. Protein concentration was measured by the Bradford method (Harlow and Lane 2006) using bovine serum albumin as a standard. Purified endoglucanase was added to 1.0% (w/v) CMC dissolved in 50 mM Tris-HCl buffer (pH 7.0). The mixture was incubated at 50 °C for 10 min. After that, reduced sugars were immediately detected by the 3,5-Dinitro salicylic acid (DNS) method (Hu et al. 2008). Specifically, 1 mL of the reaction sample was mixed with 2 mL of the DNS reagent, and the mixture was placed in a boiling-water bath for 10 min. The samples were then cooled to room temperature and absorbance at 540 nm was measured. Glucose was used as a standard for reducing sugar quantification. One unit (U) of glycosyl hydrolase activity was defined as the amount of enzyme that produced 1 μmol reducing sugar per minute. Enzyme activities of IfCelS12A on cellulosic polysaccharides substrates were determined using 500 μL of the reaction mixture, containing 100 μg mL^−1^ of IfCelS12A, and 0.5% of substrate in 50 mM of Tris–HCl buffer, at pH 7.0 (optimum pH), incubated for 50 °C for 30 min. Substrates included the following: α-cellulose, Avicel™, cellulose powder, PASC, CMC, barley b-d-glucan, xyloglucan, and xylan. To determine enzyme activity on oligosaccharides, 500 μL reaction mixture comprised of 0.5 mM oligosaccharides in 50mM Tris–HCl (pH 7.0) and IfCelS12A (300 μg mL^−1^) was incubated at 50 °C for 10 min. Hydrolysis products of polysaccharides were identified by thin-layer chromatography (TLC) as previously described (Baramee et al. 2015) using silica gel 60 plates (1.05554; 20 by 20 cm) (Merck, Darmstadt, Germany) with a mixture of *n*-butanol, acetic acid, and water (2:1:1) as a solvent (Sornyotha et al. 2007). Sugar spots were detected by heating plates to 100 °C after spraying with a reagent (4 g α-diphenylamine, 4 mL aniline, 200 mL acetone, and 30 mL 80% phosphoric acid). 

### Kinetic and product analysis of IfCelS12A

Kinetic parameters were determined by incubating IfCelS12A (0.5 μM) with cellotetraose, cellopentaose, cellullohexaose (0.25 to 3 mM), and barley b-d-glucan (0 to 3 mM) in 50 mM Tris–HCl buffer pH 7.0 at 50 °C for 10 min. The kinetic constants were determined using Lineweaver–Burk plots. Product release profiles and quantification for IfCelS12A-mediated hydrolysis were determined by high-performance liquid chromatography (HPCL model LC-20A: Shimadzu, Kyoto, Japan) on an Aminex HPX-42C column (Bio-Rad Laboratories, Hercules, CA, USA), that was operated at 85 °C with Milli-Q filtered water (EMD Millipore, Bedford, MA, USA) at a flow rate of 0.6 mL min^−1^. For most experiments, both glucose (G1) and cello-oligosaccharide (G2-G6) were used as standards. 

### Effect of NaCl on IfCelS12A activity

The effect of salt on enzyme activity was studied over a range of NaCl concentrations: 0, 5, 10, 15, 20, 25, and 30% (w/v). The stability at these concentrations was measured by incubating the purified enzyme with 10, 15, 20, 25, and 30% (w/v) NaCl at 50 °C for 24 h. Enzyme activity was measured using standard methods described above.

### Effect of metal ion and protein inhibitor on IfCelS12A activity

To observe the effect of different metal ions on IfCelS12A activity, CMC was pre-incubated with various metal ions for 1 h. The metal ions were added to achieve a final concentration of 5 mM. The same reaction without salt was used as a control. To test the inhibitory effect of protein inhibitors, IfCelS12A was incubated with EDTA, dimethyl sulfoxide (DSMO), SDS (5 mM final concentration) and glycerol, Triton X, isopropanol, and methanol (5% v/v final concentration) at 50 °C for 1 h. Enzyme activity was subsequently detected as described above. The same reaction without protein inhibitors in 10% w/v NaCl was used as an experimental control. 

### Saccharification efficiency of IfCelS12A with a cocktail under varying temperatures using MESP

The reaction mixture for testing saccharification efficiency was comprised of: 1% (w/v) of CMC with 15% (v/v) of one of three different types ILs (i.e., BMIM**-**BF4, BMIM**-**Ac, or EMIM**-**Cl**)** mixed with 3 mL of MESP-enzyme suspension at 50 °C for 24 h in a 75 mL reaction containing 20 mM Tris-malate (pH 6.0), 1 mM ATP, 25 mM MgCl_2_. This step was followed by a colorimetric assay to assess the sugar reduction efficiency. A colorimetric assay (Park and Johnson 1949) compared hydrolysis efficiency between using a cocktail from *C. thermcellum* against the same cocktail with IfCelS12A or a cocktail mix containing both IfCelS12A and MESP. After the reaction, the sample was centrifuged at 8,000 rpm for 10 min to remove residual particles of the pretreated rice straw and diluted 10 times in sterilized distilled water. 120 mL of diluted sample was mixed with 120 mL of a 50 mM Na_2_CO_3_/10 mM KCl solution and 120 mL of a 1.5 mM K_3_Fe (CN)_6_ solution. This 360 mL mixture was heated for 15 min at 100°C and added 600 mL of a 0.15% NH_4_Fe (SO_4_)_2_.12 H_2_O and 0.1% SDS/0.05 N H_2_SO_4_ solution. After incubation at room temperature for 15 min, OD_600 nm_ readings were taken using an automated plate reader. All the reactions were done in triplicate. Since, hydrolysis in BMIM**-**BF4 showed the greatest glucose released, this condition was used to examine thermal stability under moderate and high-temperature conditions (i.e., 50 °C, 60 °C, 70 °C, and 80 °C). These temperature-dependence assays followed the same protocol described above.

### Rice straw pretreated with ionic liquids (ILs)

Rice straw (RS) was dried and cut into small pieces of a 1–3 cm size using scissors and blended with a mechanical blender prior to pretreatment. Rice straw was resuspended in different IL solutions (i.e., BMIM-BF4, BMIM-Ac, and EMIM-Cl) to make 15% (v/v) suspensions. The mixtures were then incubated at 100 °C for 24 h. Resulting slurries were diluted with 50 mM Tris–HCl, pH 7.0, to obtain a final IL concentration of 15% (v/v). Pretreated solids were dried at 60 °C and ground into smaller particles using a coffee grinder and then screen sieved to gauged particle sizes of both untreated and pretreated RS. Ground particulate of ~ 150 mm was used for the reactions in this study.

### Saccharification of lignocellulosic biomass with IfCelS12A combined with MESP and enzymes from C. thermocellum

IfCelS12A, IfCelS12A in a cocktail with enzymes from *C. thermocellum*, and IfCelS12A in a cocktail with enzymes and MESP enzyme-mediated hydrolysis of cellulosic substrates (i.e., IL-pretreated RS) was conducted (with controls). The reaction mixture for saccharification was conducted with 1% (w/v) pretreated rice straw in IL slurry (or freshwater wash controls) and 3mL of charge MESP suspension at 50 °C for 24 h in 75 mL in a reaction containing 20 mM Tris-malate (pH 6.0), 1 mM ATP, 25 mM MgCl_2._ This was followed by colorimetric assays to assess sugar reduction efficiency. The colorimetric assay (Park and Johnson 1949) compared hydrolytic efficiency between IfCelS12A-only, IfCelS12A within *C. thermcellum* cocktails, and IfCelS12A-containing cocktails plus MESP conditions. Assays were completed as explained above. All the reactions were done in triplicate. 

### Statistical analysis

The data in this study are presented as mean with standard deviations provided for the experimental outcomes. Three replicates (i.e., triplicates) were used in all experiments unless otherwise indicated.

### Nucleotide sequence accession numbers

The 822 bp IfCelS12A gene sequence is deposited in the GenBank DNA database (accession no. OP904000).

## Results

### IfCelS12A amino acid sequence indicates molecular weight and primary structural features in comparison to other GH12 family hydrolases

**Fig. 1:**
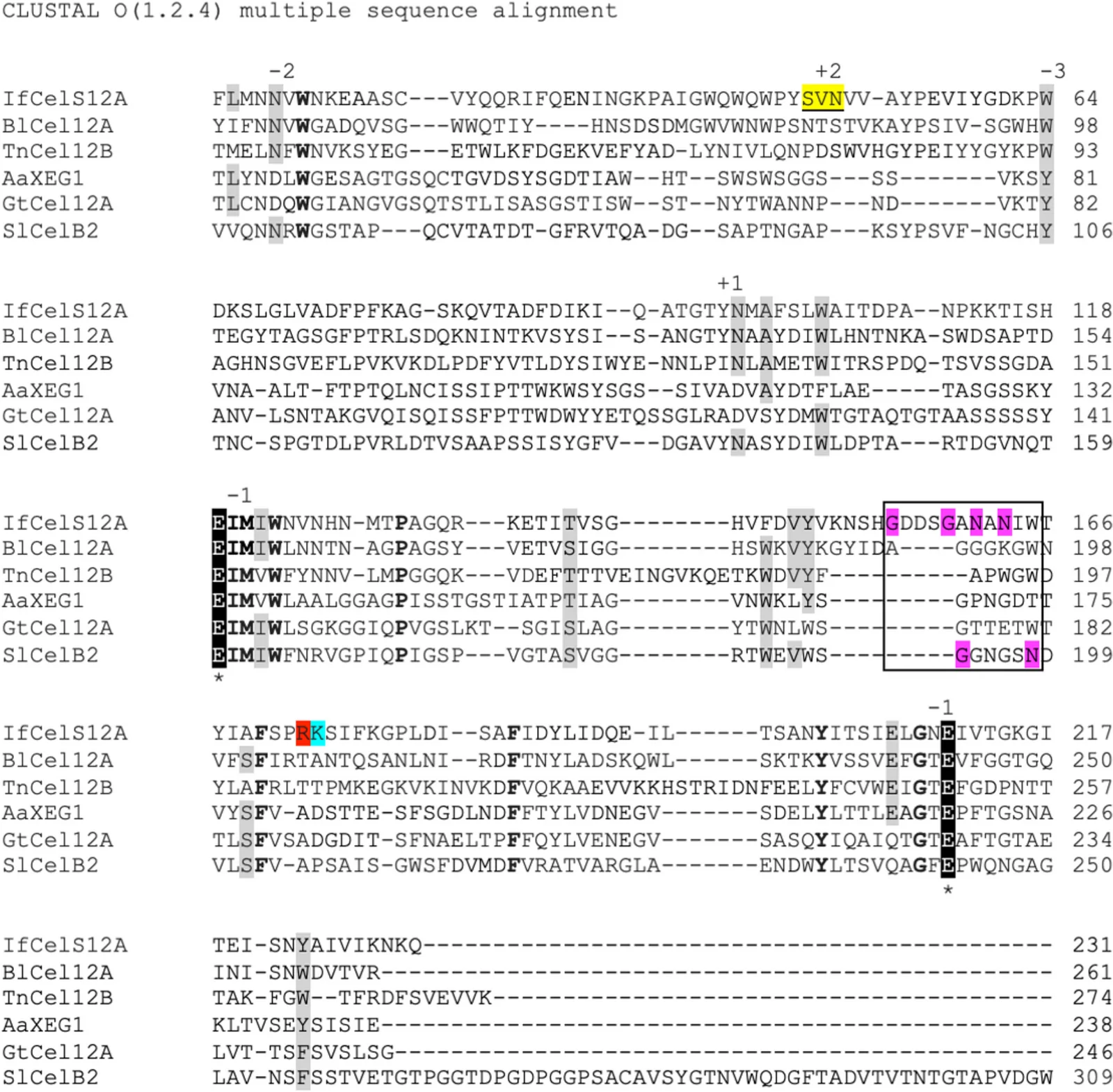
Amino acid sequence alignment of IfCelS12A compared with other GH12 hydrolases. Highly conserved residues are shown in bold*.* Other conserved residues are indicated by gray highlights. GH12 family hydrolase highly conserved catalytic domain residues are highlighted in black with an asterisk. Sequence stretches corresponding to surface loops are boxed and key loop residues (e.g., glycine, asparagine) are highlighted in violet. Amino acids with a potential role in salt tolerance are highlighted in red and turquoise. Amino acids unique to IfCelS12A with a role in substrate binding are highlighted in yellow. Enzymes that are compared: IfCelS12A (*I. fronsfrigidae*), BlCel12A (*B. lichenformis*), TnCel12B (*T. naphthopilia*), AaXEG1 (*A. aculeatus*), GtCel12A (*G. trabeum*), SlCelB2 (*Strepomyces lividans*)

Sequence analysis of the *IfCelS12A* gene reveals that the ~ 822 nucleotide gene encodes a protein of ~ 273 amino acids with a predicted molecular weight of ~ 30.1 kDa (Supplemental Fig. S1) and a characteristic modular structure consisting of an upstream signaling peptide domain followed by the GH12 family conserved region (Supplemental Fig. S2). Alignment of *I. fonsfrigidae* SP3-1 IfCelS12A against other enzymes within the GH12 family of hydrolases reveals characteristic amino acids at key locations within the primary structure (Fig. 1). Specifically, previously reported conserved residues are present within the protein sequence, including Glu210, Met121, Asn13, Trp64, Asn98, Ser48, Val49, Asn50, Glu119, and Glu210 (Boyce and Walsh 2018; Akram and ul Haq 2020; Sulzenbacher et al. 1999; Pauly et al. 1999; Oh et al. 2019). Other conserved residues of unknown function are also there, such as Trp15, Trp123, Ile120, Pro131, Glu208, Phe170, Phe186, and Tyr201. In contrast to conserved amino acids, there are other positions that show conspicuous variability (Fig. 1, grey highlighted). Notably, IfCelS12A only exhibits ~ 28% overall sequence identity to the other hydrolases used in the sequence comparison. Nonetheless, when compared to GtCel12A from *G. trabeum* (ATCC 11539), SlCelB2 from *S. lividans*, TnCel12B2 from *T. naphthopilia* strain RKU-10, AaXEG from *A. aculeatus*, and BlCel12A from *B. lichenformis*, phylogenetic analysis of the conserved domain reveals that IfCelS12A is nested within the family of GH12 hydrolases (Supplemental Fig. S3).

### Substrate specificity, kinetic analysis, and catalytic efficiency of purified IfCelS12A

**Fig. 2:**
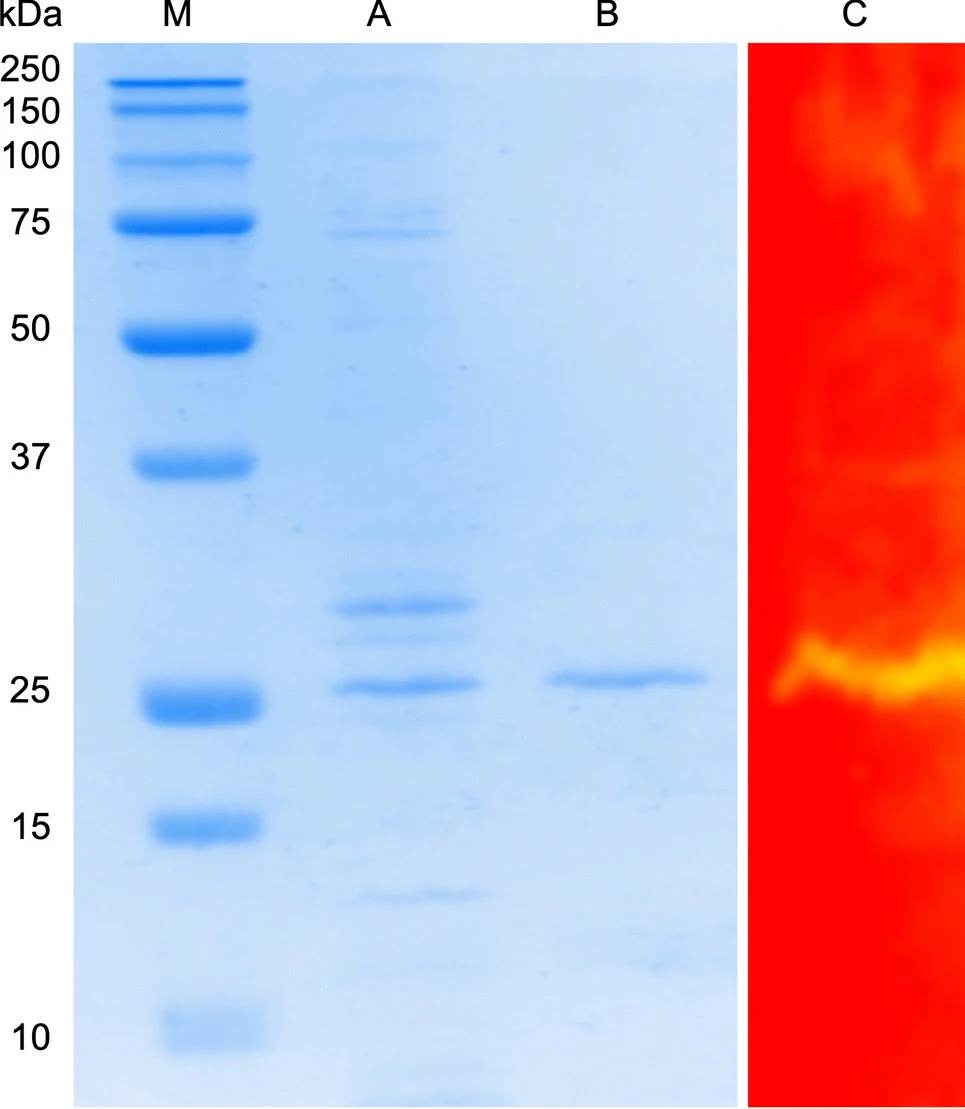
Zymogram and SDS-PAGE (10%) analysis of protein fractions after overexpression of recombinant IfCelS12A in *E. coli* BL21 (DE3) cells. 10–250 kDA protein ladder [Lane M]. Crude lysate (diluted 1:50) [Lane A]. Purified fraction using His-Tag column [Lane B]. Zymogram of IfCelS12A showing enzymatic activity [Lane C]

IfCelS12A was analyzed using protein gel electrophoresis (i.e., SDS-PAGE, 10%). Crude lysate from an *E. coli*-based overexpression system was diluted 1:50 for band clarification (Fig. 2, Lane A). Purification was done using an N-terminal His tag on the recombinant IfCelS12A (i.e., His Trap™ FF column, Sigma-Aldrich, St. Louis, USA) to highlight the target protein band (Fig. 2, Lane B). For both crude lysate and the purified eluate, a ~ 30 kDa band representing the recombinant IfCelS12A was observed. Zymogram analysis was performed to demonstrate that IfCelS12A exhibits hydrolase activity (Fig. 2, Lane C). In addition, to general hydrolase activity, specific activity of IfCelS12A was examined for different carbohydrate substrates (Table 1). IfCelS12A exhibits higher levels of activity (> 75 U/mg) on CMC (~ 221 U/mg) and PASC (~ 164 U/mg) than other substrate. However, significant IfCelS12A enzymatic activity is also observed (~ 36 U/mg) with barley β-d-glucan as the substrate. No activity was observed on a-cellulose, cellulose powder, Avicel, xyloglucan, birchwood, b-1,3-glucan, or oat spelt xylan (Table 1, top rows). In addition to testing activity on polysaccharides, several oligosaccharide substrates were tested. IfCelS12A exhibits high activity on cellotetraose, cellopentaose, and cellohexaose (> 75 U/mg); however, no hydrolysis was observed for cellobiose or cellotriose (Table 1, bottom rows). IfCelS12A kinetic parameters were also examined using Michaelis-Menten and Lineweaver-Burk plots (Table 2). Specifically, *k*_*cat*_ and *K*_*M*_ were examined for four substrates: cellotetraose, cellopentaose, cellohexaose, and barley β-d-glucan. A 1.4 to 1.7-fold increase in *k*_*cat*_* /K*_*M*_ was observed for purified oligosaccharides over more complex substrates (e.g., barley β-d-glucan), shows a *k*_*cat*_*/K*_*M*_ value of 0.08 mM^−1^ s-^1^ (Table 2).

**Table 1:** The specific activity of IfCelS12A on different carbohydrate substrates

Substrates	Specific activity (U/mg protein)
Polysaccharide^a^	
Carboxymethyl cellulose (CMC, 1% w/v)	221.16 ± 0.543
Phosphoric acid swollen cellulose (PASC, 1% w/v)	164.48 ± 0.731
a-Cellulose (1% w/v)	−
Cellulose powder (1% w/v)	−
Avicel (1% w/v)	−
Xyloglucan (tamarind seed, 1% w/v)	−
Birchwood xylan (1% w/v)	−
Oat spelt xylan (1% w/v)	−
Barley β-d-glucan (β-1,3;1,4-glucan, 0.5% w/v)	35.79 ± 0.118
β-1,3-Glucan (0.5% w/v)	−
Oligosaccharide^b^	
Cellobiose (0.5 mM)	−
Cellotriose (0.5 mM)	−
Cellotetraose (0.5 mM)	89.98 ± 0.006
Cellopentaose (0.5 mM)	77.43 ± 0.003
Cellohexaose (0.5 mM)	95.82 ± 0.005

– Enzyme is not active on this substrate

^a^1 U_P_ = μmol cellobiose or xylobiose production/min mg IfCel12A

^b^1 U_S_ = μmol substrate degradation/min mg IfCel12A

**Table 2:** IfCelS12A kinetics on select substrates (50 °C, 50 mM Tris–HCl buffer pH 7.0)

Substrates	*K*_*M*_ (mM)	*k*_*cat*_ (S^−1^)	*k*_*cat*_* /K*_*M*_ (mM^−1^S^−1^)
Cellotetraose	0.25	0.37	1.48
Cellopentaose	0.27	0.36	1.30
Cellohexaose	0.39	0.50	1.26
Barley b-d-glucan	0.04	0.51	0.08

### The mode of action of IfCelS12A reveals the differences from the other GH12 family

**Fig. 3:**
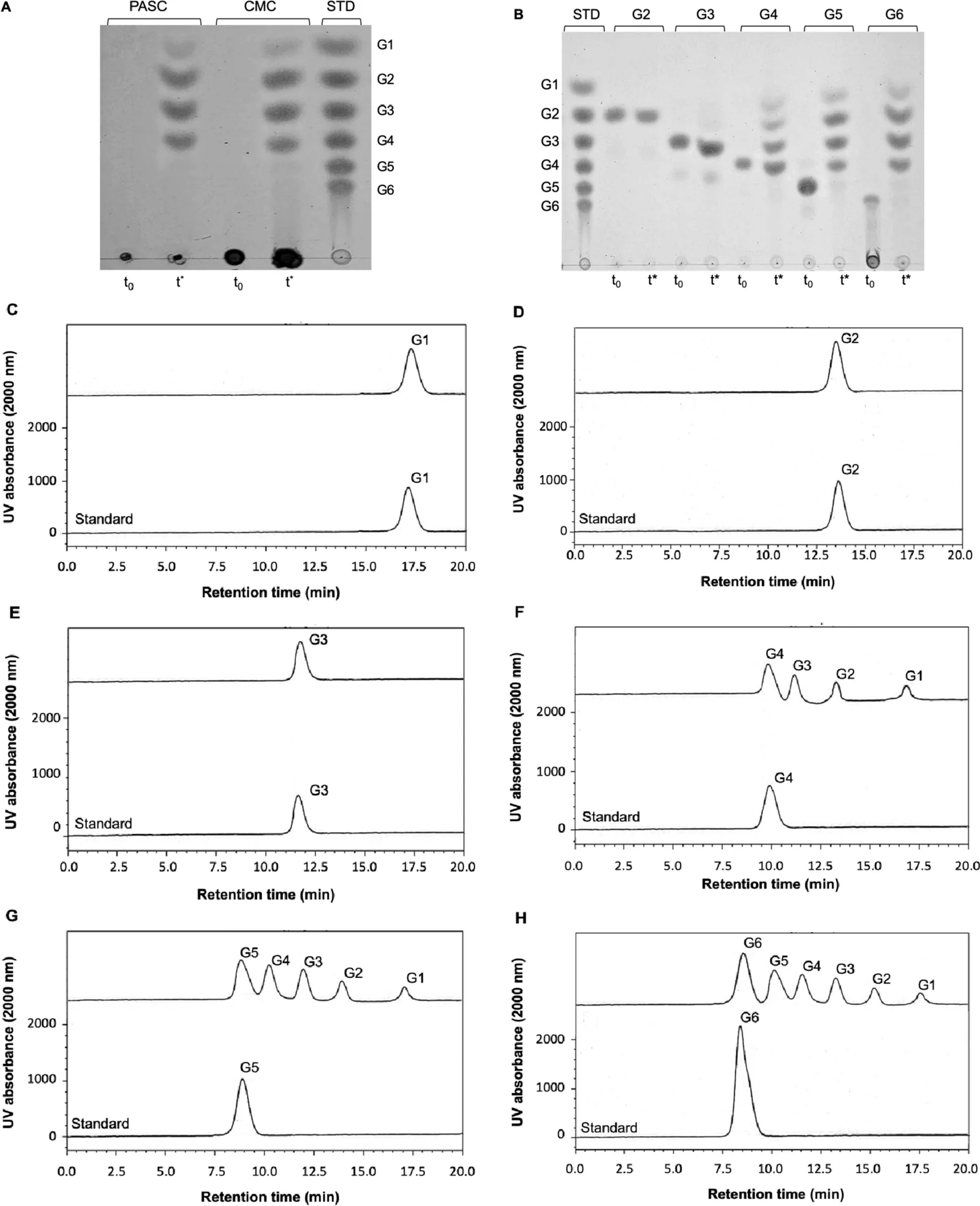
Glucose release products from IfCelS12A hydrolysis. **A** Thin-layer chromatography (TLC) analysis of IfCelS12A-mediated hydrolysis reveals release products from PASC and CMC after substrate-enzyme incubation at 50 °C for 30 min. Lane 1, PASC at *t*_0_ (cello-oligosaccharide negative control). Lane 2, PASC hydrolysis products at endpoint (t^*^). Lane 3, CMC at *t*_0_ (cello-oligosaccharide negative control). Lane 4, CMC hydrolysis products at endpoint (t^*^). Lane 5, standards for oligo-saccharides (positive control). (G1, glucose; G2, cellobiose; G3, cellotriose; G4, cellotetraose; G5, cellopentaose; G6, cellohexaose; PASC, phosphoric acid-swollen cellulose; CMC, carboxyl methyl cellulose). **B** TLC analysis of IfCelS12A-mediated hydrolysis reveals release products from cello-oligosaccharides. Lane 1, oligo-saccharide standards (positive controls; standard, STD) (G1, glucose; G2, cellobiose; G3, cellotriose; G4, cellotetraose; G5, cellopentaose; G6, cellohexaose). Lane 2, cellobiose at *t*_0_ (negative control). Lane 3, cellobiose hydrolysis products at endpoint (t^*^). Lane 4, cellotriose at *t*_0_ (negative control). Lane 5, cellotriose hydrolysis products at endpoint (t^*^). Lane 6, cellotetraose at *t*_0_ (negative control). Lane 7, cellotetraose hydrolysis products at endpoint (t^*^). Lane 8, cellopentaose at *t*_0_ (negative control). Lane 9, cellopentaose hydrolysis products at endpoint (t^*^). Lane 10, cellohexaose at *t*_0_ (negative control). Lane 11, cellohexaose hydrolysis products at endpoint (t^*^). (No enzyme added to negative controls). **C**–**H** High-performance liquid chromatography (HPLC) analyses IfCelS12A-mediated hydrolysis of different cello-oligosaccharides reveals spectra for release products after enzymatic digestion of substrate after 30 min of incubation

The mode of action of IfCelS12A CMC, PASC, and cello-oligosaccharides was examined by analyzing hydrolysis release products (Fig. 3). Both thin-layer chromatography (TLC) and high-performance liquid chromatography (HPLC) were used to show the types of release products (Fig. 3A) and quantity (Fig. 3B), respectively. Products from PASC and CMC include cellobiose, cellotriose, and cellotetraose (Fig. 3B). IfCelS12A appears to rapidly hydrolyze cellohexaose, cellopentaose, and cellotetraose (Fig. 3B, F, G, and H). IfCelS12A-mediated hydrolysis of cellohexaose results in the release of cellotetraose, cellotriose, cellobiose, and glucose (Fig. 3B, Lane G6 and Fig. 3H). Hydrolysis of cellopentaose results in the release of cellotetraose, cellotriose, cellobiose, and glucose (Fig. 3B, Lane G5, and Fig. 3G). IfCelS12A hydrolysis of cellotetraose results in the production of cellotriose, cellobiose, and glucose (Fig. 3B, Lane G4, and Fig. 3F). IfCelS12A does not appear to hydrolyze cellotriose or cellobiose (Fig. 3B, Lanes G2 and G3; Fig. 3D and E). Cumulatively, these data indicate the mode of action for IfCelS12A-mediated hydrolysis of oligosaccharides (see Fig. 3B, F, G, and H ). Note that for all substrates only low amounts of glucose are released in comparison to other products (e.g., cellobiose).

### Glucose release assays demonstrate the salt tolerance profile for IfCelS12A catalytic function

**Fig. 4:**
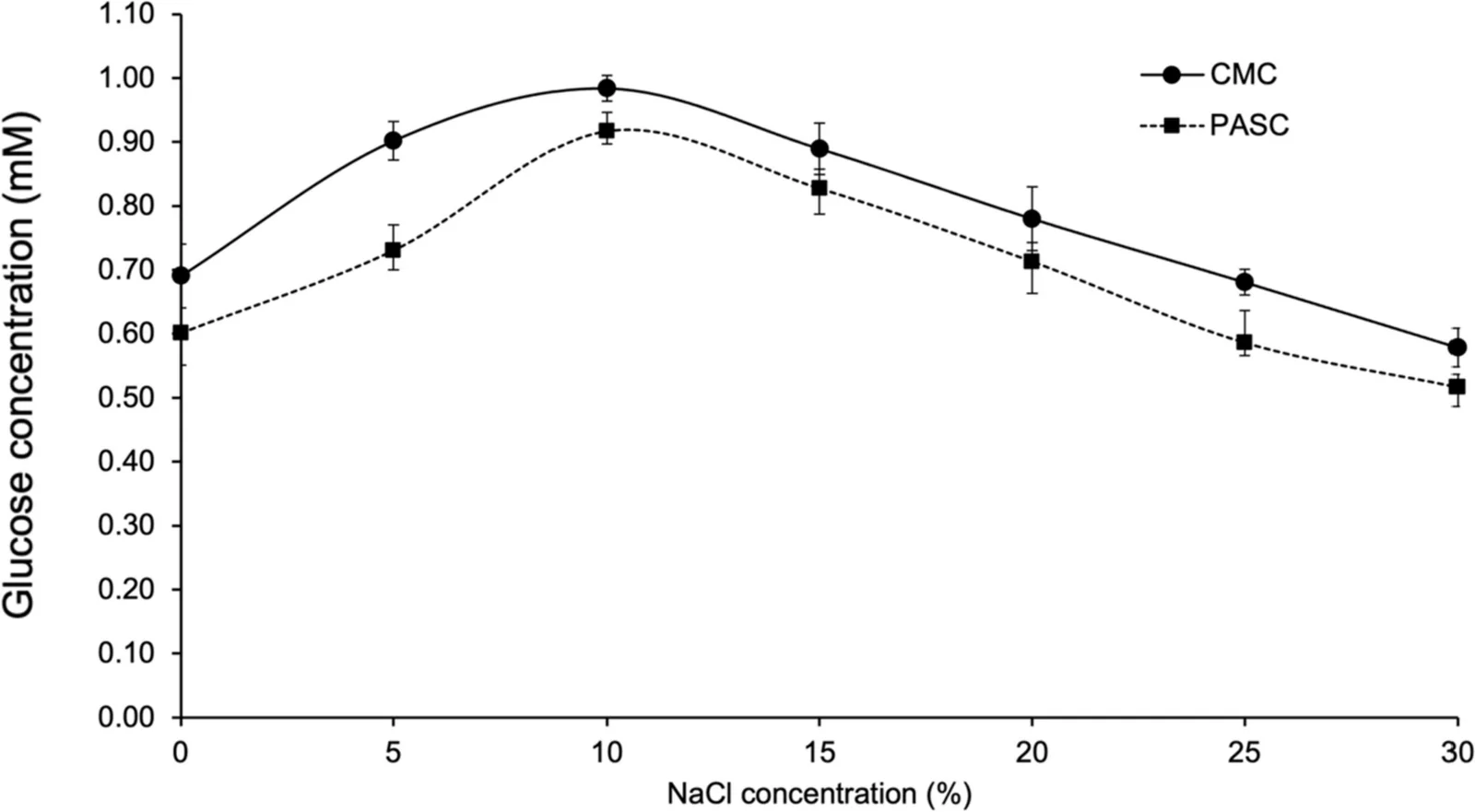
Enzymatic efficiency of IfCelS12A as a function of salt (NaCl) concentration during glucose release. Enzymatic activity of IfCelS12A on two substrates: CMC and PASC—in the presence of increasing salt (i.e., NaCl) concentrations with 0% w/v NaCl serving as the baseline against which all other conditions are measured. IfCelS12A activity on CMC (diamond studded line) shows maximum catalytic efficiency at 10% w/v NaCl with an effect of NaCl on the purified IfCelS12A. NaCl profile was determined at various NaCl range from 0 to 30% (w/v) using with 1% (w/v) CMC as substrate by incubating purified IfCelS12A (50 ng/mL) in Tris buffer of pH 7.0 for 30 min. The highest activity was observed at 10% NaCl (w/v), which was defined as 100% activity to calculate the relative activity

To test the enzymatic efficiency of IfCelS12A under hypersaline conditions, purified IfCelS12A activity was examined over a broad range of salt concentrations (0–30% w/v NaCl). Assay data demonstrate that glucose release from substrate (i.e., CMC and PASC) during IfCelS12A-mediated hydrolysis steadily increases in 5–15% w/v NaCl with optimal activity at 10% w/v NaCl (Fig. 4). This profile is consistent for both CMC and PASC substrates. In addition, our results indicate that IfCelS12A is functional at hypersaline concentrations up to 30% w/v NaCl, albeit less active than the baseline/control. This shows the salt tolerance of this *I. fronsfrigidae* GH12 family hydrolase.

### IfCelS12A enzymatic efficiency differs depending upon the type of metal ion present in the solution

The effect of metal ions on IfCelS12A enzymatic activity was examined using CMC as the substrate. Glucose release activity was compared to baseline controls (i.e., 0% salt concentration = 100% activity) for IfCelS12A-mediated hydrolysis of CMC in the presence of different metal salts at 5 mM (Table 3). Glucose release assay data show that IfCelS12A-mediated hydrolysis was highest in solutions containing: CaCl_2_, NaCl, MnSO_4_, and BaCl_2_. Significant activity, although less than baseline, was also observed for solutions containing: AgSO_4_, FeCl_2_, NH_4_Cl, and MgCl_2_. Metals detrimentally impacting IfCelS12A activity (i.e., < 50% relative activity) include: NiCl_2,_ KCl, ZnSO_4_, and HgSO_4_ (Table 3, upper rows).

**Table 3:** Effect of metal ions, chemical reagents, and organic solvents on IfCelS12A from *I. fonsfrigidae* strain SP3-1

Metal ions (5 mM)	Relative activity (%)*
No additive	100.0
NiCl_2_	16.7
AgSO_4_	83.3
FeCl_2_	66.7
NH_4_Cl	88.9
CaCl_2_	138.9
MgCl_2_	72.2
NaCl	150.0
KCl	21.3
MnSO_4_	144.4
BaCl_2_	116.7
ZnSO_4_	11.1
HgSO_4_	22.2
Solvents (5% v/v)	Relative activity (%)
SDS	50.0
EDTA	44.4
DSMO	83.3
Glycerol	72.2
Triton X	100.0
Isopropanol	88.9
Methanol	61.1

^*^To analyzed effect of IfCelS12A with various metal ions, chemicals, and solvents, enzyme assays were performed in Tris–HCl buffer (pH 7.0) at 50°C, under standard assay conditions using CMC as the substrate. All reactions were carried out in triplicate

### The effect of chemical reagents and organic solvents demonstrates the behavior of IfCelS12A

To examine the effects of organic solvents and other reagents on IfCelS12A catalytic efficiency, glucose release data from CMC were collected using different additives at 5% (v/v) (Table 3, lower rows). Glucose release assay data indicate that compared to the no additive control, only mild impact on IfCelS12A-mediated hydrolysis occurs when isopropanol, dimethylsulfoxide (DMSO), or glycerol are added to solution. Only a 10.1%, 16.7%, and 27.8% decrease in glucose release efficiency was observed in solutions containing: isopropanol, DMSO, and glycerol, respectively (Table 3, lower rows). Notable decreases (> 30%) in IfCelS12A catalytic efficiency on CMC are observed when sodium dodecyl sulfate (SDS), methanol, or ethylenediaminetetraacetic acid (EDTA) is added to solution (Table 3, lower rows).

### IfCelS12A enhances saccharification efficiency when used as part of an enzyme cocktail

**Fig. 5:**
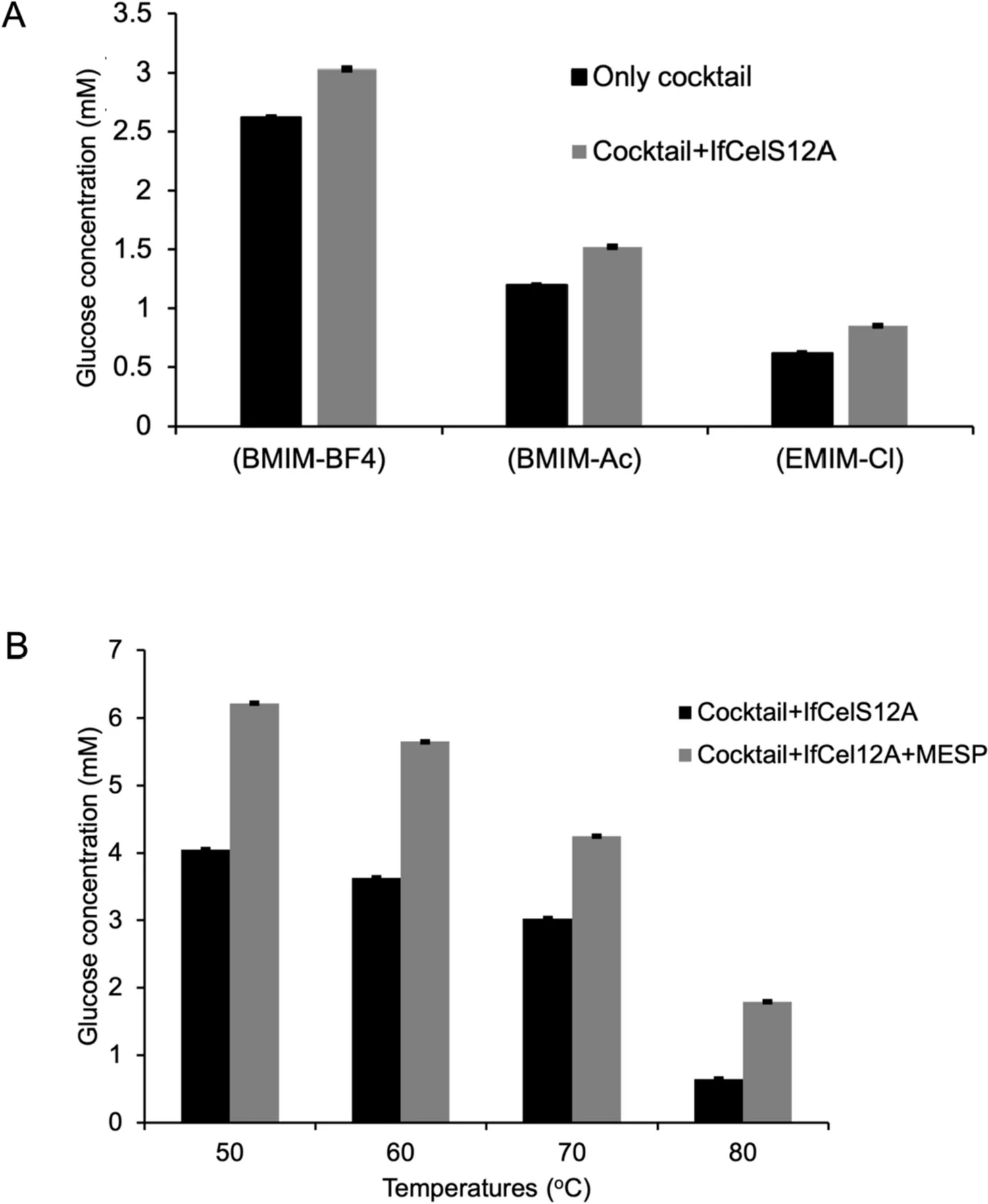
Glucose release assay for cellulolytic enzyme cocktail versus cocktail containing IfCelS12A. **A** Glucose release from CMC degradation mediated by a cellulolytic enzyme cocktail (i.e., CelD, CelK, XynA, BglA) versus the same cocktail with IfCelS12A added. Reactions were performed in different ILs. IfCelS12A enhances saccharification of CMC when used as part of an enzyme cocktail in different ILs as shown by glucose release from CMC employing the *C. thermocellum* enzyme cocktail only (black bars) versus glucose release from CMC using the same cocktail augmented with IfCelS12A from *I. fronsfrigidae* (gray bars). ILs used in assays include: 1-butyl-3-methylimidazolium tetrafluoroborate (BMIM-BF4), 1-butyl-3-methylimidazolium acetate (BMIM-Ac), or 1-ethyl-3-methylimmidazolium chloride (EMIM-Cl). **B** Glucose release from CMC degradation mediated by a cellulolytic enzyme cocktail (i.e., CelD, CelK, XynA, BglA) versus the same cocktail with IfCelS12A and the thermotolerant archael based MESP at different temperatures in BMIM-BF4 at pH 7. IfCelS12A-containing cocktail with MESP enhances saccharification of CMC when compared to IfCelS12A-containing cocktail only (i.e., sans MESP) as demonstrated by glucose release from CMC using IfCelS12A-containing cocktail only (black bars) compared to glucose release from CMC using IfCelS12A-containing cocktail with MESP (gray bars)

Since data demonstrate the ability of IfCelS12A to bind longer chain oligosaccharides (e.g., cellotetraose and cellohexaose), IfCelS12A was tested as a component within an enzyme cocktail to determine if it enhances saccharification efficiency. Specifically, IfCelS12A was combined with a standard cocktail of cellulolytic enzymes used in the conversion of cellulosic substrates that includes: CelD (endoglucanase), CelK (exoglucanase), XynA (xylanase), and BglA (b-glucanase) derived from the thermophilic anaerobic bacterium *C. thermocellum* (see Ceballos et al. 2014). Three different IL solutions were used in a CMC slurry (Fig. 5A). Under all three conditions glucose release from CMC was enhanced when IfCelS12A was added to the cocktail.

### Saccharification using IfCelS12A cocktail is enhanced under varying temperature using MESP

Cocktails composed of enzymes from *C. thermocellum* are reported to enhance cellulosic substrate degradation under temperatures that are higher than what may be viable for mesophilic enzymes (Mitsuzawa et al. 2009; Ceballos et al. 2014). To test whether IfCelS12A, IfCelS12A with MESP, or IfCelS12A added to a cocktail with *C. thermocellum* enzymes functions under different temperatures (i.e., 50 °C, 60 °C, 70 °C, and 80 °C), glucose release from CMC was studied (Supplemental Fig. S4). It has also been reported that cellulolytic enzymes in the presence of or bound to an engineered archaeal chaperonin-based mobile enzyme sequestration platform (MESP) enhances enzyme-mediated hydrolysis of lignocellulosic substrates (Mitsuzawa et al. 2009; Ceballos et al. 2014). Therefore, an MESP-containing cocktail was also tested (Fig. 5B, grey bars). Data show that glucose release in cocktail plus IfCelS12A declines but is stable from 50 to 70 °C; however, a fourfold drop in efficiency from baseline (i.e., 50 °C) is observed at 80 °C (Fig. 5B, black bars). When MESP complexes are added to the cocktail mixture and then into the CMC slurry, an approximate 30% increase in glucose release is observed across all temperature conditions (Fig. 5B, grey bars).

### Breakdown of IL-pretreated rice straw is enhanced when using IfCelS12A cocktail and MESP

**Fig. 6:**
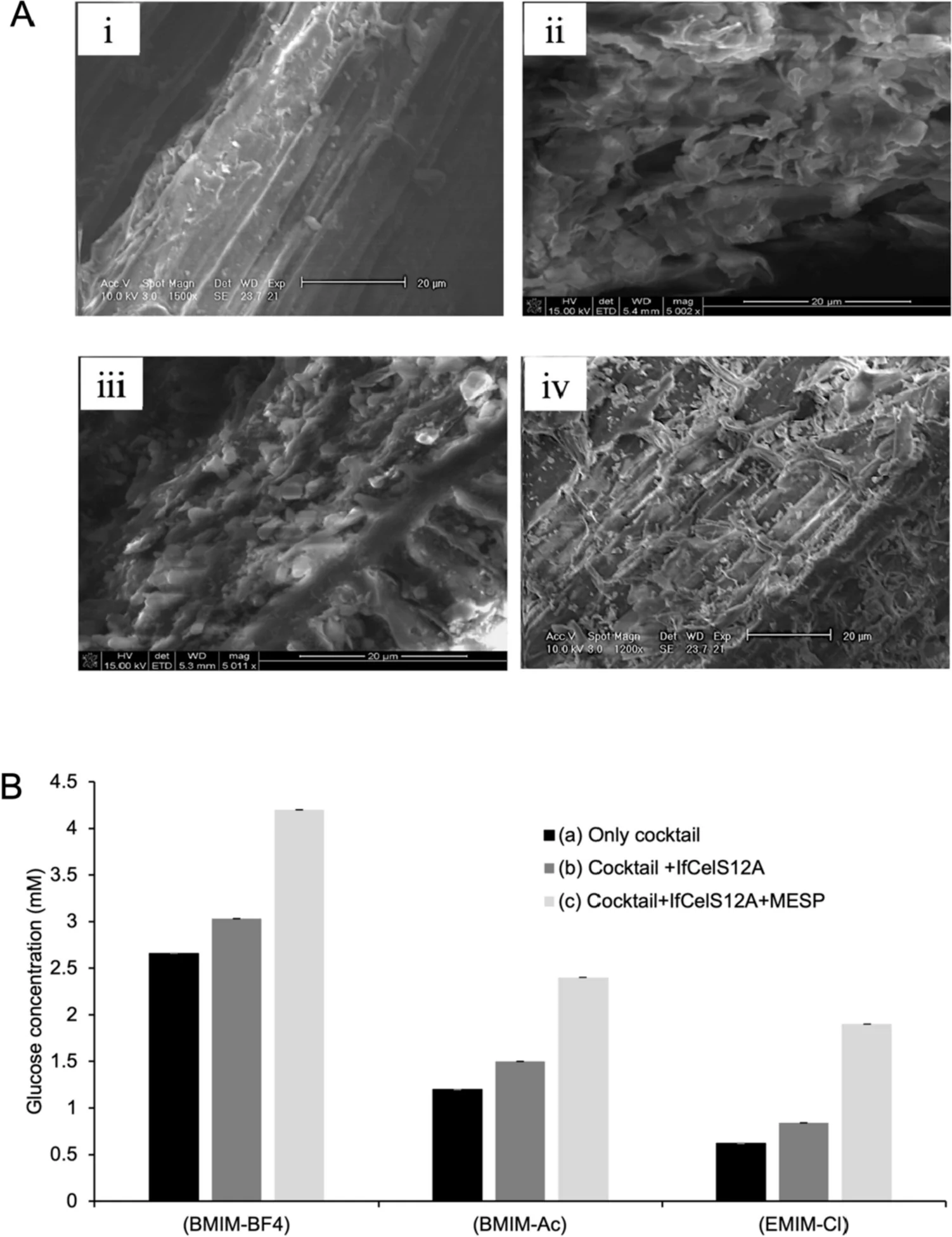
Ionic liquid pretreatment of rice straw. **A** Scanning electron micrographs (SEM) of RS after pretreatment with different ILs. **i** SEM of untreated RS (control) reveals a higher density, ordered, and more compact (i.e., unswollen) feedstock structure. **ii** RS treated with BMIM-BF4 exhibits observable material swelling, deconstructed architecture, and frayed/chipped microstructure. **iii** RS treated with BMIM-Ac also shows material swelling and deconstructed architecture with some chipping/fraying. **iv** EMIM-Cl treatment of RS exhibits some swelling (but less than other IL pretreatments) shows deconstructed architecture with substantial chipping (compared to other IL pretreatments). **B** Glucose release from RS treated with different ILs using different enzyme regimens reveals difference in glucose release: cocktail composed only of *C. thermocellum* cellulolytic enzymes (black bars); cocktail augmented with IfCelS12A (dark grey bars); and IfCelS12A-containing cocktail plus MESP (light grey bars). IL slurries tested are: BMIM-BF4, BMIM-Ac, and EMIM-Cl. Enhancement of enzymatic degradation of RS with IfCelS12A plus MESP results in the highest glucose release

To examine if the advantages of IfCelS12A hydrolysis of simpler substrates extrapolate to commonly used lignocellulosic feedstocks pre-treated in IL, three IL solutions were used to pretreat rice straw (RS) prior to conducting glucose assays. Scanning electron microscopy (SEM) reveals that RS treated for 24 h (i.e., overnight) with 1-butyl-3-methylimidazolium tetrafluoroborate (BMIM-BF4), 1-butyl-3-methylimidazolium acetate (BMIM-Ac), or 1-ethyl-3-methylimmidazolium chloride (EMIM-Cl) results in substrate deconstruction in the form of structural swelling and fraying when compared to untreated controls (Fig. 6Ai). Qualitatively, BMIM-BF4 appears to readily expose internal structures of RS particulate/fiber (Fig. 6Aii). To test enzymatic efficiency of IL-pretreated RS using IfCelS12A and MESP, glucose release was examined with the following: (a) *C. thermocellum* enzyme cocktail-only; (b) cocktail plus IfCelS12A; and (c) cocktail plus IfCelS12A with MESP added (Fig. 6B). Data show that glucose release in all enzyme configurations is greatest in BMIM-BF4 (Fig. 6B, left). Across all IL slurry types, an increase in glucose release (~ 12–26%) is observed when IfCelS12A is added to cocktails of *C. thermocellum* enzymes (Fig. 6B). A notable enhancement in enzyme-mediated glucose release (30–44%) is observed across all IL slurry types when MESP are added to the mixture (Fig. 6B, light grey). Glucose release from RS in a cocktail of *C. thermocellum* enzymes, IfCelS12A, and MESP appear to be optimal in BMIM-BF4 (15% v/v) at 50 °C, and pH 7.

## Discussion

Discovery and characterization of cellulolytic enzymes with unique attributes, such as stability at elevated temperatures and tolerance to low pH, is an active area of research in applied microbiology (e.g., Elleuche et al. 2015; Husain 2017; Thapa et al. 2020; Dadwal et al. 2021; Gan et al. 2022). For this study, we characterized an endoglucanase (i.e., IfCelS12A) derived from the halophile *I. fonsfrigidae* strain SP3-1 isolated from a hypersaline pond in Thailand (Heng et al. 2022).

### IfCelS12A is a unique member within the GH12 family of glycoside hydrolases

An indicator that IfCelS12A is a member of the GH12 family of glycoside hydrolases are the conserved amino acids found in the primary structure that are characteristic of this family of enzymes. IfCelS12A features conserved residues at positions—Glu210, Met121, Asn13, Trp64, Asn98, Ser48, Val49, and Asn50—which are known to be involved in substrate binding (Boyce and Walsh 2018; Akram and ul Haq 2020). IfCelS12A also possesses conserved residues at positions—Glu119 and Glu210—which are reported to be involved in substrate hydrolysis (Sulzenbacher et al. 1999; Pauly et al. 1999; Oh et al. 2019). Other key residue positions in the alignment show some variability (see Fig. 1, grey highlighted); however, many of the substitutions contain amino acids with functional similarity. For example, at position 122, IfCelS12A presents isoleucine as observed in Bl12CelA, Gt12CelA, and SlCelB2; however, in corresponding positions, TnCel12B and AaXEG1 express Val155 and Val136, respectively. Despite the alternative amino acids, both isoleucine and valine are hydrophobic. Likewise, at position 146, IfCelS12A presents phenylalanine but other GH12 hydrolases show tryptophan at the corresponding position. Again, both of these alternative amino acids are hydrophobic in nature indicating potential conservation of function at this position (see Fig. 1). Despite the identical and functionally similar amino acids at key locations among the compared hydrolases, there is a low overall sequence identity of IfCelS12A to other GH12 hydrolases. Indeed, IfCelS12A only exhibits ~ 28% overall sequence identity to the other hydrolases examined, which may suggest that IfCelS12A is not a GH12 family hydrolase. However, analyses of the core functional domains (e.g., substrate binding motif) clearly set IfCelS12A within the GH12 family.

Although phylogenetic analyses (and enzyme function assays) indicate that IfCelS12A is a GH12 glycoside hydrolases, sequence analyses and enzyme activity assays also reveal features that distinguish IfCelS12A from other GH12 hydrolases.

Moreover, several of the noted differences in the IfCelS12A amino acid sequence may result in functional consequences. For example, Arg173 (Fig. 1, red highlighted) and Lys174 (Fig. 1, turquoise highlighted), which is a unique feature of IfCelS12A, may contribute to the salt tolerance of this enzyme (Mevarech et al. 2000). IfCelS12A also exhibits key amino acid differences in the flexible region of the protein. This includes a GDDSG residue run and two asparagine residues (i.e., Asn161 and Asn163) not found in other GH12 hydrolases (Fig. 1, boxed and violet highlighted). This unique motif changes the nature of the roof of the active site in the enzyme (Dorival et al. 2020). Furthermore, in the catalytic domain, which is bounded by two glutamate residues (see Fig. 1, black highlighted with asterisks), IfCelS12A features the notable presence of an arginine residue (R173) and a lysine residue (K174). This motif has been previously reported (Mevarech et al. 2000) to facilitate formation of salt-bridges that bind sodium and chloride with lysine residues directly interacting with chloride ions. Although it is beyond the scope of this study, a detailed analysis of three-dimensional structure and molecular dynamics modeling would indicate whether this profile, which is not found in other GH12 family hydrolases, alters the active site “roof” in IfCelS12A (Sandgren et al. 2013). It is also suggested that the presence of Arg173 (Fig. 1, red highlighted) and Lys174 (Fig. 1, turquoise highlighted) may endow this enzyme with higher salt tolerance compared to other GH12 family hydrolases. Another unique feature of IfCelS12A is the SVN run of amino acids within the active site at positions 48–50, which is not found in any of the comparator GH12 hydrolases (Fig. 1, yellow highlighted). The influence that this alternative triad has on substrate binding is unknown. Cumulatively, these distinct features of IfCelS12A along with similarities to the comparators indicate that IfCelS12A is indeed a GH12 hydrolase but potentially with different catalytic abilities. This was verified through a series of enzyme assays, which demonstrated IfCelS12A activity differs from other GH12 hydrolases. Similar to most other GH12 family hydrolases, IfCelS12A is unable to decompose cellobiose into two glucose molecules (Oh et al. 2019; Kuntothom and Cairns 2020). IfCelS12A does act on cellulosic and hemicellulosic substrates, including CMC, PASC, mannan, xyloglucan, barley-b-d-glucan, Avicel™, filter paper, and xylan (Wittmann et al. 1994; Bok et al. 1998; Pauly et al. 1999; Karlsson et al. 2002; Damásio et al. 2012; Zhu et al. 2019; Oh et al. 2019; Kuntothom and Cairns 2020; Akram and ul Haq 2020; Ma et al. 2021). However, there are also noted differences between IfCelS12A and other GH12 hydrolases in terms of substrate range, binding affinity for select oligosaccharides, and release product profile, whereas many GH12 hydrolases act on cellotriose, cellotetraose, cellopentose, and cellohexaose, IfCelS12A only acts on cellotetraose, cellopentose, and cellohexaose (Supplemental Table S1). Thus, IfCelS12A has a narrower substrate range for oligosaccharides compared to some other GH12 hydrolases, including GtCel12A (Oh et al. 2019) and TbCel12A (Kuntothom and Cairns 2020). In terms of binding affinity and turnover rate, IfCelS12A exhibits lower catalytic power (i.e., lower *k*_*cat*_/*K*_*M*_) than some hydrolases (e.g., TrCel12A) on cellotetraose and cellopentaose (Karlsson et al. 2002), while exhibiting higher *k*_*cat*_*/K*_*M*_ than others, such as HiCel12A (Schou et al. 1993) on these same substrates (see Table 4).

**Table 4:** Comparison of IfCelS12A enzyme kinetics with other GH12 hydrolases

Substrates	Sources	Kinetic parameter			References
		*K*_*M*_ (mM)	*k*_*ca*t_ (s^−1^)	*k*_*cat*_*/K*_*M*_ (mM^−1^ s^−1^)	
Cellotetraose	IfCelS12A	250	0.37	0.0013	Present study
	TrCel12A	430	0.80	0.002	Karlsson et al. 2002
	HiCel12A	1300	0.0019	1.5 × 10^–6^	Schou et al. 1993
Cellopentaose	IfCelS12A	270	0.36	0.0013	Present study
	TrCel12A	230	14	0.06	Karlsson et al. 2002
	HiCel12A	130	0.0016	1.2 × 10^–5^	Schou et al. 1993

In addition to differences in enzyme kinetics, IfCelS12A has a different product release profile than other GH12 hydrolases when acting on cellotetraose, cellopentaose, or cellohexaose (Supplemental Table S2). GtCel12A (Oh et al. 2019) and TbCel12A (Kuntothom and Cairns 2020) hydrolysis of cellotetraose results in a notable release of cellobiose, while cellotriose and glucose are not stoichiometrically favored release products. In contrast, IfCelS12A-based hydrolysis of cellotetraose results in the release of glucose, cellotriose, and cellobiose (Supplemental Fig. S5, Supplemental Table S2). This release of glucose is notable since IfCelS12A hydrolysis of cellopentaose yields cellotetraose, a product which is not released by either GtCel12A (Oh et al. 2019) or TbCel12A (Kuntothom and Cairns 2020). IfCelS12A also releases cellotetraose from cellohexaose (Supplemental Fig. S5, Supplemental Table S2).

### In addition to salt tolerance IfCelS12A is active under a suite of adverse reaction conditions

Since IfCelS12A is derived from an extreme halophile, demonstrated tolerance under high salt conditions is anticipated. However, IfCelS12A also performs well in vitro under a range of elevated temperatures (see Supplemental Fig. S6), in the presence of different metals (see Table 3), and with organic solvents or detergents (see Table 3). Specifically, IfCelS12A exhibits enhanced activity (i.e., increased glucose release) at elevated temperatures from 37 to 60 °C when compared to room temperature (i.e., 25 °C) controls. Catalytic efficiency declines precipitously after 60 °C to ~ 50% of control at 70 °C and 80 °C (Supplemental Fig. S6). Not surprisingly, IfCelS12A maintains high catalytic efficiency in the presence of sodium chloride (NaCl) and calcium chloride (CaCl_2_). However, data also demonstrate high activity in solutions containing barium chloride (BaCl_2_) and manganese sulfate (MnSO_4_) with appreciable activity even in the presence of silver sulfate (AgSO_4_), magnesium chloride (MgCl_2_), and ferrous chloride (FeCl_2_). Catalytic function in the presence of various metals/metal salts opens the possibility for enzyme use in bioremediation applications. Interestingly, our data also show high relative activity in the presence of ammonium chloride (NH_4_Cl), which presents the potential use of IfCelS12A in enzyme cocktails after ammonia fiber expansion (AFEX) pretreatment of lignocellulosic substrates.

Remarkably, IfCelS12A retains activity in some solvents and detergents that may be expected to reduce enzymatic activity or denature enzymes. For example, in the presence of Triton X-100, there is no appreciable loss in IfCelS12A enzymatic activity. In DMSO, 83% of activity is observed. Although low concentrations of DMSO do not result in complete denaturation or complete loss of IfCelS12A activity, DMSO may suppress enzyme flexibility by exposing hydrophobic residues (Roy et al. 2012). Even in low concentrations (5% v/v) of sodium dodecyl sulfate (SDS), IfCelS12A remains active but at only 50% relative activity (see Table 3). IfCelS12A retains hydrolytic activity in the presence of alcohols, such as isopropanol (89%) and methanol (61%) but at reduced levels. Even low concentrations of isopropanol (i.e., 5% v/v) may render enzymes less rigid (and more prone to unfolding), which can increase structural instability, thus accounting for decreased activity (Kamal et al. 2013; Tsuzuki et al. 2003). In the more extreme cases of enzyme degradation, SDS and methanol present harsher environments, resulting in increased enzyme denaturation. IfCelS12A does not perform well (< 50%) in the presence of ethylenediaminetetraacetic acid (EDTA), a chelating agent. This is likely due to the need for divalent cations to stabilize protein structure for enzymatic function. As a chelating agent, EDTA also has a strong negative impact on enzymatic activity by disrupting the structural stability of the enzyme via sequestration of stabilizing cations (Nisar et al. 2022). Glycerol only mildly impacts IfCelS12A enzymatic activity, which is important for long-term storage of purified enzyme in 5–10% glycerol. The ability of IfCelS12A to retain activity in organic solvents and detergents is advantageous when considering the use of IfCelS12A as part of an enzyme cocktail. Enzymes that function across a broader temperature range and that are resilient in the presence of metals, organic solvents, and detergents permit substrate specificity optimization, non-polar substrate solubilization, reversal of the thermodynamic equilibrium of hydrolysis reactions, water-dependent side reaction removal, and elimination of microbial contamination (Mohtashami et al. 2019).

### IfCelS12A emerges as candidate for enzyme cocktails designed to deconstruct cellulosic substrates

Ionic liquids are often used as a pretreatment or solution base for breaking down cellulosic feedstock and reducing complex carbohydrates (i.e., cellulose, hemicellulose) to simpler sugars (Kumar et al. 2021; Kuntapa et al. 2021; Shukla et al. 2023). Generally, enzymes exhibit reduced efficiency under high salt conditions (Albarracín et al. 2011; Sinha and Datta 2016; Kumawat et al. 2022). The discovery and characterization of IfCelS12A offer an option for enhancing enzyme-mediated cellulosic substrate deconstruction under hypersaline conditions. Our data reveal that on cellulosic substrates, such as PASC and CMC, introducing IfCelS12A as part of an enzyme cocktails results in higher yields of released glucose (see Fig. 5A). Recently, our lab has developed and patented a mobile enzyme sequestration platform (MESP) that protects enzymes under adverse reactions conditions (e.g., elevated temperature, mild pH flux) while stabilizing or enhancing enzymatic activity (Ceballos et al. 2015; Furr et al. 2021). IfCelS12A activity is also enhanced in the presence of MESP in solution (Fig. 5B) as part of an enzyme cocktail system for degrading cellulosic substrate. Although IfCelS12A releases sufficient glucose for testing enzyme efficiency in glucose release assays, it would not be the main enzyme for glucose release in an enzyme cocktail. Indeed, IfCelS12A is more effective in the presence of cellobiose hydrolases (e.g., b-glucosidases). However, its action on longer chain oligosaccharides (e.g., cellotetraoses, cellopentaoses) makes it a candidate for cocktail design.

### Results from IfCelS12A-containing cocktails extrapolate to raw feedstock preparations

Glucose release assays on PASC and CMC exclude confounds associated with pretreated and enzymatically degrading raw lignocellulosic feedstock (e.g., effects of lignin, mechanical disruption, particle size). Therefore, the potential for IfCelS12A to serve as a complementary component of an enzyme regimen requires a validation of the effects on processed feedstock. In this study, rice straw (RS) was pretreated with ILs (i.e., (BMIM-BF4), (BMIM-Ac), and (EMIM-Cl)) to determine the potential benefits of having IfCelS12A incorporated into an enzyme cocktail for hydrolysis of lignocellulosic substrates (Fig. 6A). Our data show that adding IfCelS12A as a component in an enzyme cocktail in the absence (Fig. 6B, grey) or presence (Fig. 6B, light grey) of MESP technology enhances sugar reduction as measured by glucose release in vitro. Addition of IfCelS12A to enzyme cocktails composed of CelD, CelK, XynA, and BglA from *C. thermocellum* offers an enhanced ability to breakdown longer chain oligosaccharides that, generally, have lower binding or limited substrate specificity with other enzymes in the cocktail. Although more detailed studies need to be conducted to examine the potential synergistic effects of using specific cellulolytic, hemicellulolytic, and ligninolytic enzymes in conjunction with IfCelS12A, we can conclude that IfCelS12A is a viable candidate for enzyme cocktail design for deconstructing lignocellulosic substrates, particularly in IL. The characterization of salt-tolerant enzymes from halophiles that act on cellotriose would also complement this work and provide additional options for cocktail design and optimization.

## Supplementary Information

Below is the link to the electronic supplementary material.

Supplementary file1 (PDF 855 KB)

## Data Availability

The authors declare that all the data supporting the findings of this study are available within the article and its supplementary information files: nucleotide and amino acid sequence of IfCelS12A; the modular structure of IfCelS12A; the phylogenetic tree of IfCelS12A with other members in the GH12 family; IfCelS12 pH profile; IfCelS12A temperature profile; and comparison of the product release of IfCelS12A and other GH12 family. All the data generated or analyzed during this study are included in the published article. *Iocasia fronsfrigidae* strain SP3-1 is deposited at the Thailand Institute of Scientific and Technological Research Culture Collection (TISTR) as well as at the Korean Collection for Type Cultures (KCTC) under accession numbers TISTR 2992 and KCTC 25333, respectively.
