# Supplementary material for: Hydrolysis of ionic liquid–treated substrate with an Iocasia fonsfrigidae strain SP3-1 endoglucanase
Source: Appl Microbiol Biotechnol. 2024 Jan 8;108:63. doi: 10.1007/s00253-023-12918-1 (PMC10774164; doi:10.1007/s00253-023-12918-1)
Supplement: Supplementary file 1 [file 253_2023_12918_MOESM1_ESM.pdf]

Applied Microbiology and Biotechnology

Hydrolysis of ionic liquid treated substrate with an *Iocasia fonsfrigidae* strain SP3-1 endoglucanase

Sobroney Heng[1,7], Sawannee Sutheeworapong[2], Chinnapong Wangnai[3], Verawat Champreda[4], Akihiko Kosugi[5], Khanok Ratanakhanokchai[1,6], Chakrit Tachaapaikoon[1,6*], and Ruben Michael Ceballos[7,8,9*]

[1]School of Bioresources and Technology, King Mongkut's University of Technology Thonburi, Bangkok 10150, Thailand
[2]Systems Biology and Bioinformatics Laboratory, Pilot Plant Development and Training Institute, King Mongkut's University of Technology Thonburi, Bangkok 10150, Thailand
[3] Pilot Plant Development and Training Institute, King Mongkut's University of Technology Thonburi, Bangkok, Thailand
[4] National Center for Genetic Engineering and Biotechnology, 113 Thailand Science Park, Paholyothin Road Klong Luang, Pathumthani 12120, Thailand
[5] Biological Resources and Post-harvest Division, Japan International Research Center for Agricultural Sciences, Ibaraki, Japan
[6] Excellent Center of Enzyme Technology and Microbial Utilization, Pilot Plant Development and Training Institute, King Mongkut's University of Technology Thonburi, Bangkok 10150, Thailand
[7] Department of Biological Sciences, University of Arkansas, Fayetteville, AR, USA 72701
[8] Arkansas Center for Space & Planetary Sciences, University of Arkansas, Fayetteville, AR, USA 72701
[9] Molecular and Cell Biology Department, University of California – Merced, Merced, CA, USA 95343

*** Correspondence:**
Ruben Michael Ceballos
rceballos@ucmerced.edu

Supplementary Data

```
1      ______M__I__M__L__N__D__D__Y__F__L__M__N__N__V__W__N__K__E__
121    TGGGGAATGATCATGCTGAATGACGATTACTTTTTAATGAATAACGTCTGGAATAAAGAG
19     A__A__S__C__V__Y__Q__Q__R__I__F__Q__E__N__I__N__G__K__P__A__
181    GCTGCCTCCTGCGTTTACCAGCAAAGAATCTTTCAAGAGAACATCAATGGCAAACCAGCA
39     I__G__W__Q__W__Q__W__P__Y__S__V__N__V__V__A__Y__P__E__V__I__
241    ATTGGCTGGCAATGGCAATGGCCGTATAGTGTTAATGTAGTTGCTTATCCAGAGGTTATT
59     Y__G__D__K__P__W__D__K__S__L__G__L__V__A__D__F__P__F__K__A__
301    TATGGCGATAAGCCTTGGGACAAGTCACTGGGACTGGTGGCAGACTTTCCATTTAAAGCC
79     G__S__K__Q__V__T__A__D__F__D__I__K__I__Q__A__T__G__T__Y__N__
361    GGTTCTAAGCAAGTGACTGCTGATTTTGATATTAAGATACAAGCTACCGGTACTTATAAT
99     M__A__F__S__L__W__A__I__T__D__P__A__N__P__K__K__T__I__S__H__
421    ATGGCTTTTTCACTATGGGCAATCACCGATCCTGCTAATCCGAAAAAAACCATCAGCCAT
119    E__I__M__I__W__N__V__N__H__N__M__T__P__A__G__Q__R__K__E__T__
481    GAAATCATGATCTGGAATGTCAATCATAATATGACACCTGCGGGCCAAAGGAAAGAGACT
139    I__T__V__S__G__H__V__F__D__V__Y__V__K__N__S__H__G__D__D__S__
541    ATTACGGTTAGTGGGCACGTTTTCGATGTTTATGTAAAAAACAGTCATGGTGACGATTCG
159    G__A__N__A__N__I__W__T__Y__I__A__F__S__P__R__K__S__I__F__K__
601    GGAGCAAACGCCAATATTTGGACTTATATTGCGTTTTCCCCTCGAAAATCAATCTTCAAA
179    G__P__L__D__I__S__A__F__I__D__Y__L__I__D__Q__E__I__L__T__S__
661    GGGCCACTGGATATCAGCGCTTTCATTGATTATCTCATCGATCAAGAAATATTAACAAGC
199    A__N__Y__I__T__S__I__E__L__G__N__E__I__V__T__G__K__G__I__T__
721    GCAAATTATATCACTAGTATCGAACTTGGAAATGAAATTGTCACCGGAAAGGGAATCACA
219    E__I__S__N__Y__A__I__V__I__K__N__K__Q__*__
781    GAAATCAGTAATTATGCGATTGTTATTAAAAATAAACAATAA
```

Note: predicted molecular weight of IfCelS12A is ~30.1 kDa (calculated above sequences using a Peptide and Protein Molecular Weight Calculator (https://www.aatbio.com/tools/calculate-peptide-and-protein-molecular-weight-mw on 07/18/2023).

Supplementary Fig. S1 Nucleotide and amino acid sequences of *IfCelS12A*. The signal peptide is underlined. The letter in the box of ATG represents the start codon and the translational stop codon (TAA) is exhibited by an asterisk.

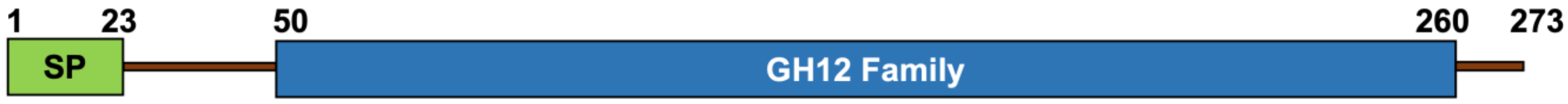

Supplementary Fig. S2 Modular structure of the IfCelS12A gene. The modular structure of *IfCelS12A* shows a signal peptide sequence upstream from the primary sequence of the glycoside hydrolase, which is characteristic of the GH12 family of enzymes. Positions 1-23 represent the commonly found GH12 enzyme signal peptide sequence. Positions 23-50 and 260-273 are non-specific hits. Positions 50-260 are the core sequence for glycoside hydrolase of the GH12 family of enzymes.

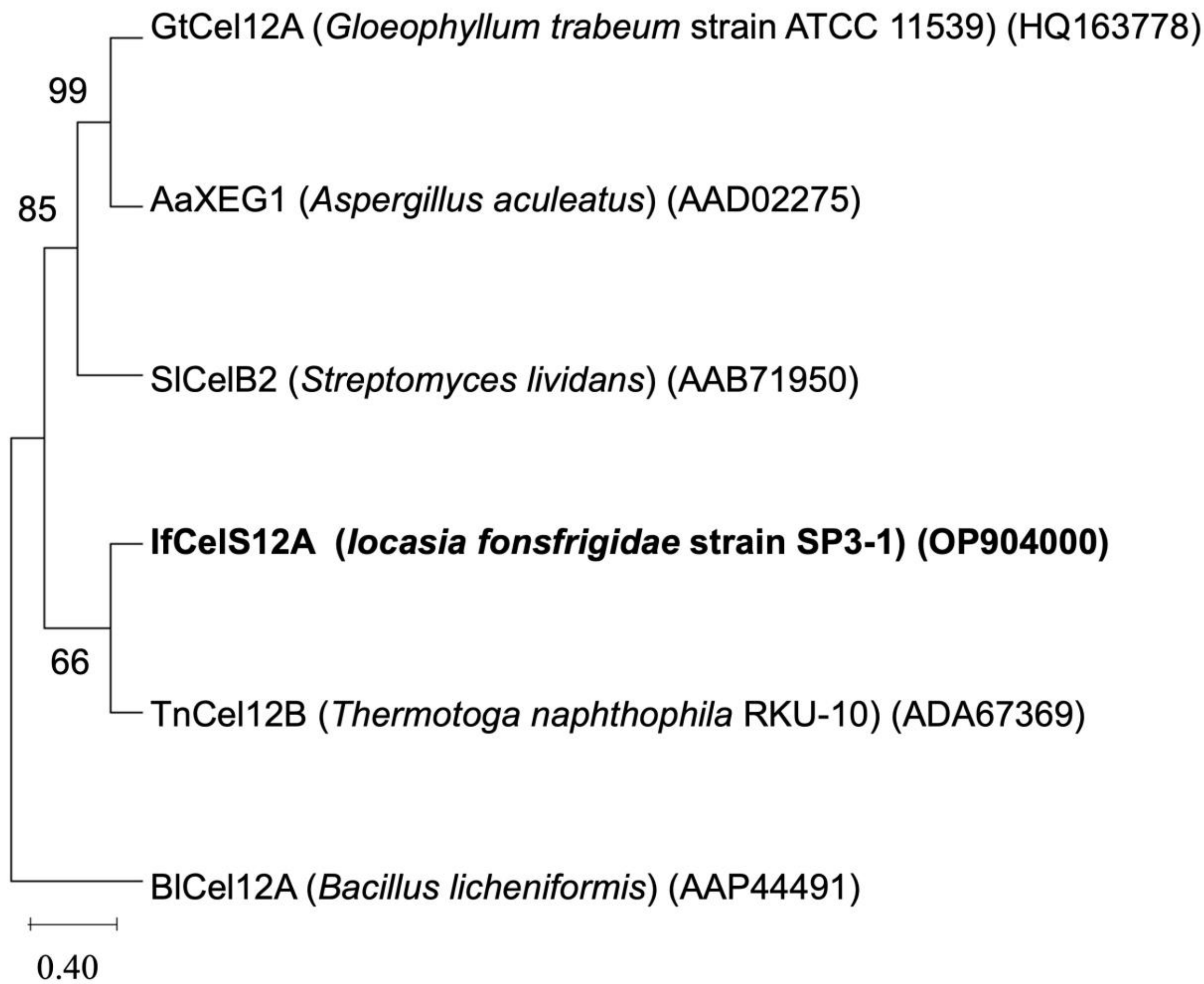

Supplementary Fig. S3 Phylogenetic analysis of the catalytic domain of IfCelS12A. A phylogenetic analysis of IfCelS12A with other GH12 family members: *G. trabeum* (GtCel12A) strain ATCC 11539 (HQ163778), *T. naphthophila* (TnCel12B2) RKU-10 (ADA67369), *A. aculeatus* (AaXEG) (AAD02275), *S. lividans* (SlCelB2) (AAB71950), *B. licheniformis* (BlCel12A) (AAP44491) – reveals that the catalytic domain of IfCelS12A is nested within a cluster of previously describe GH12 hydrolases. (The phylogenetic was constructed using the neighbor-joining method and a bootstrap analysis of 1000 replicates in the MEGA 11 Program).

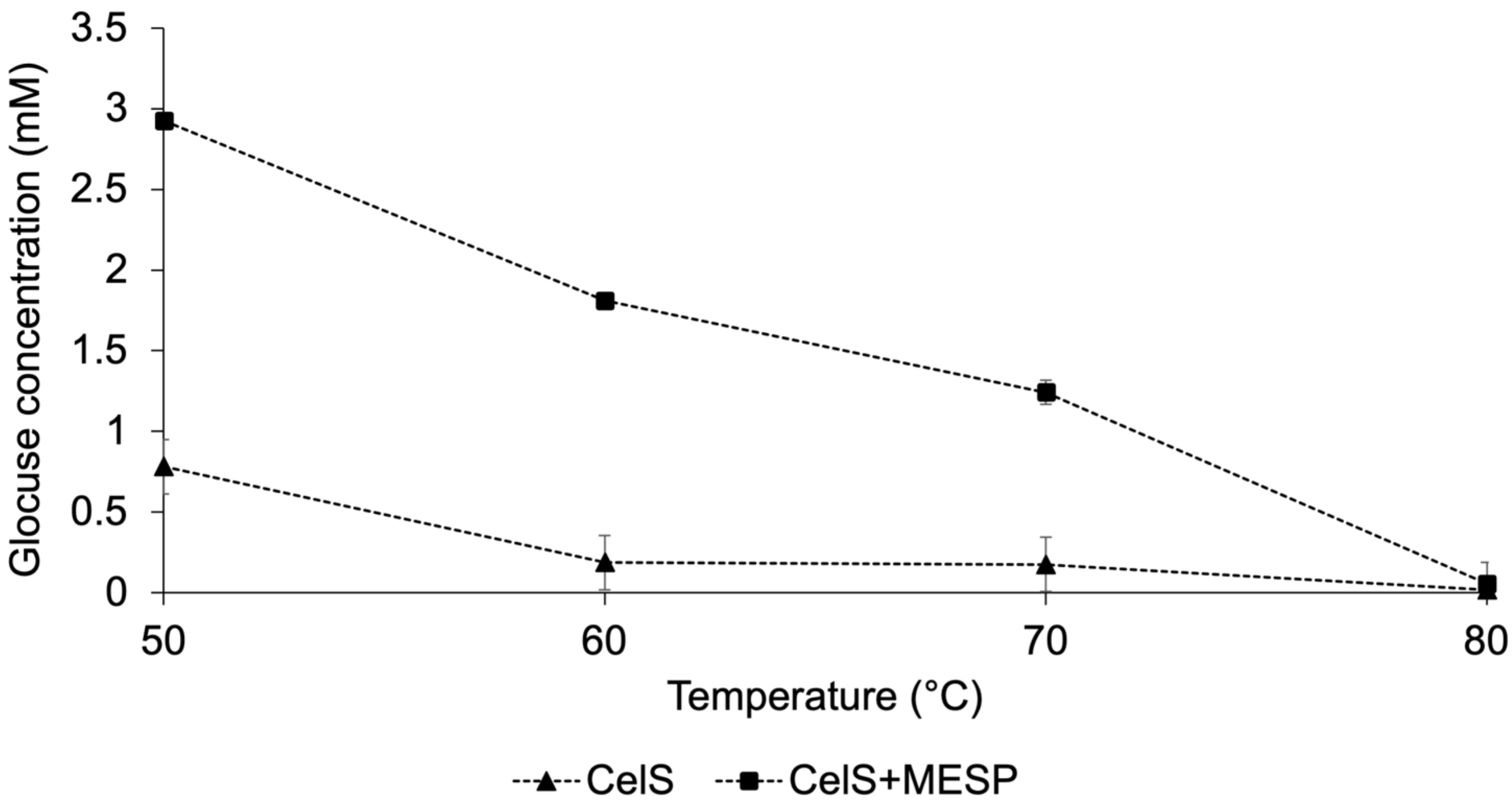

Supplementary Fig. S4 IfCelS12A hydrolytic activity in the absence or presence of MESP at elevated temperatures. Optimal enzymatic activity is observed at 50°C for both IfCelS12A alone and IfCelS12A with MESP. However, a noted increase in hydrolytic activity on the substrate (i.e., CMC) is noted when MESP is employed. Furthermore, IfCelS12A in the presence of MESP promotes increase glucose release at higher reaction temperatures. Incubation time for each temperature trial was 60 min on 1.0% (w/v) CMC.

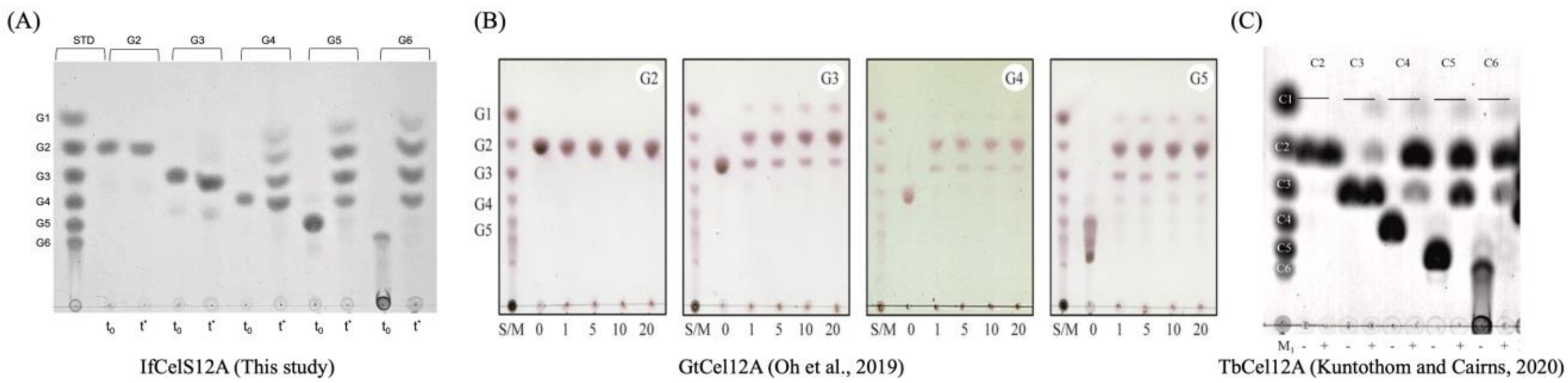

Supplementary Fig. S5 Comparison of TLC product release data between IfCelS12A data and previously reported GH12 hydrolase activity. **A** IfCelS12A hydrolysis of cellooligosaccharide (present study). **B** TLC yields from GtCel12A-mediated hydrolysis of cellooligosaccharides (Oh et al. 2019). **C** TLC profile from TbCel12A-mediated hydrolysis of cellooligosaccharide (Kuntothom and Cairns 2020).

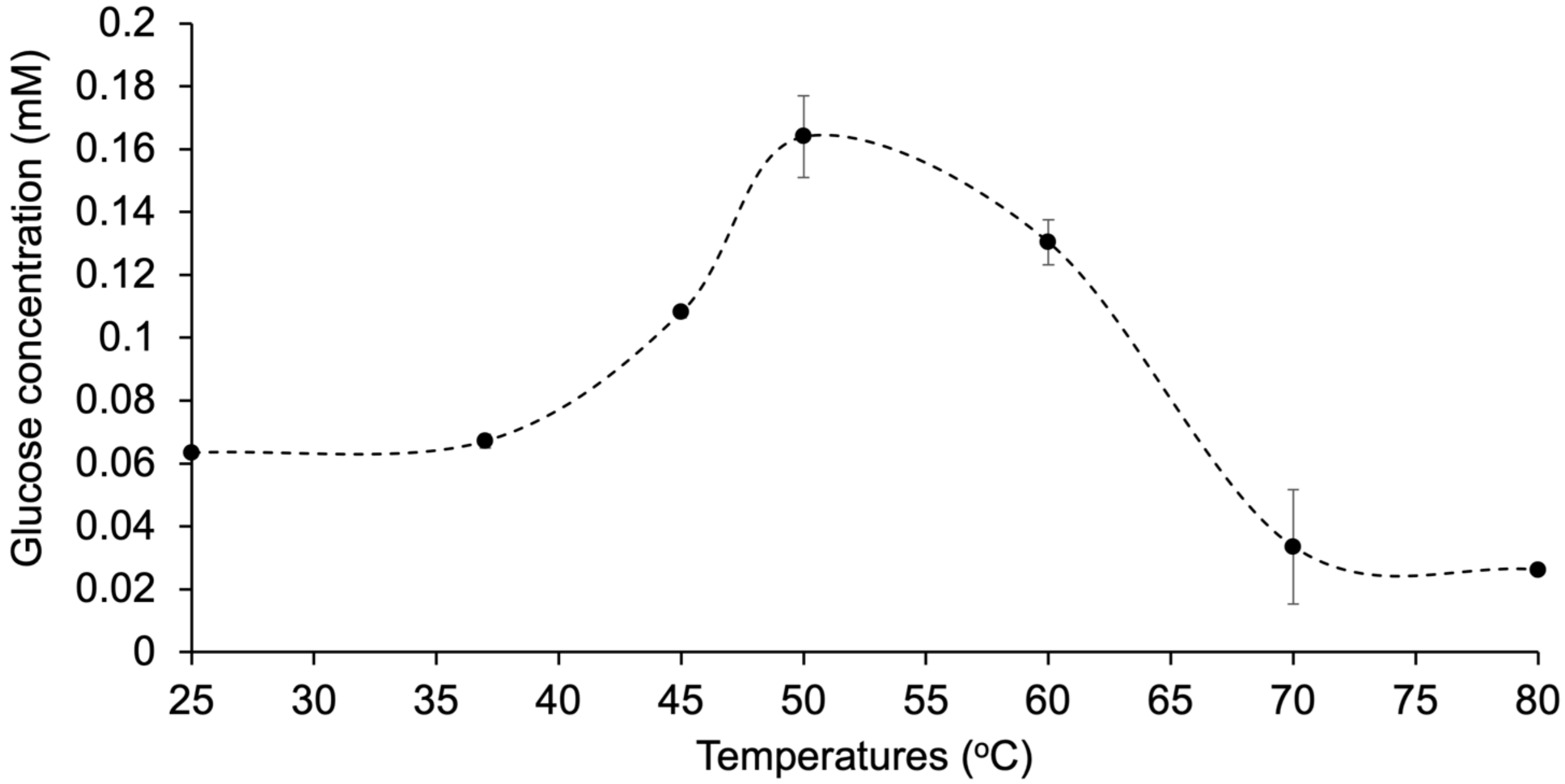

Supplementary Fig. S6 Temperature profile for IfCelS12A-mediated hydrolysis of substrate. IfCelS12A-mediated hydrolysis of CMC was examined at different temperatures. Optimal temperature for hydrolysis is 50°C. Incubation time for each temperature trial was 30 min on 0.5% (w/v) CMC.

Table S1 Comparison of hydrolysis ability on cellooligosaccharide of IfCelS12A with other GH12 family

Substrate	Hydrolysis ability (binding)		
	IfCelS12A (present study)	GtCelS12A (Oh et al. 2019)	TbCel12A (Kuntothom and Cairns 2020)
Cellobiose (G_2)	–	–	–
Cellotriose (G_3)	–	+	+
Cellotetraose (G_4)	+	+	+
Cellopentose (G_5)	+	+	+
Cellohexaose (G_6)	+	+	+

(–) : no hydrolytic activity; (+): hydrolysis detected

Table S2 Comparison of the product release from cellooligosaccharide of IfCelS12A with other GH12 family

Substrate	Product release		
	IfCelS12A (present study)	GtCelS12A (Oh et a. 2019)	TbCel12A (Kuntothom and Cairns 2020)
Cellotriose (G_3)	ND	$G_1 << G_2 = G_3$	$G_1 << G_2 = G_3$
Cellotetraose (G_4)	$G_1 << G_2 < G_3 = G_4$	$G_1 << G_2 >> G_3 = G_4$	$G_1 << G_2 >> G_3 = G_4$
Cellopentose (G_5)	$G_1 << G_2 \leq G_3 > G_4 = G_5$	$G_1 << G_2 >> G_3 >> G_4 = G_5$	$G_1 << G_2 \geq G_3 = G_5$
Cellohexaose (G_6)	$G_1 << G_2 \leq G_3 > G_4 = G_6$	NR	$G_1 << G_2 >> G_3 = G_6$

ND: not determined ; NR: not reported; <<: less than; ≤: less than or equal; >>: greater than; ≥ : greater than or equal
